# Metformin Mitigates Diabetes-Driven Renal Senescence via Immunomodulation and the FABP4/FOXO1 Axis

**DOI:** 10.3390/ph18121834

**Published:** 2025-12-01

**Authors:** Nawal M. Alrasheed, Lama A. Almuthanbi, Rana R. Alotaibi, Asma S. Alonazi, Maha A. Alamin, Tahani K. Alshammari, Dalal A. Alkhelb, Anfal F. Bin Dayel, Hatun A. Alomar, Doaa M. Elnagar, Nouf M. Alrasheed

**Affiliations:** 1 Department of Pharmacology and Toxicology, College of Pharmacy, King Saud University, Riyadh 11451, Saudi Arabia; nalrasheed@ksu.edu.sa (N.M.A.); lamaalmuth@outlook.com (L.A.A.); 441200367@student.ksu.edu.sa (R.R.A.); aaloneazi@ksu.edu.sa (A.S.A.); mahaali@ksu.edu.sa (M.A.A.); talshammary@ksu.edu.sa (T.K.A.); dalkhelb@ksu.edu.sa (D.A.A.); abindayel@ksu.edu.sa (A.F.B.D.); hetalomar@ksu.edu.sa (H.A.A.); 2 Department of Zoology, College of Science, King Saud University, Riyadh 11451, Saudi Arabia; elnagard1@yahoo.com

**Keywords:** metformin, fatty acid-binding protein 4, forkhead box protein 1, immunosenescence, immunometabolic, diabetic nephropathy

## Abstract

**Background:** Diabetic nephropathy (DN) accelerates renal aging through chronic inflammation and metabolic dysregulation; however, the role of metformin in this process remains incompletely understood. This study investigated whether metformin attenuates diabetes-driven renal senescence through the modulation of the fatty acid-binding protein 4 (FABP4)/forkhead box protein O1 (FOXO1) axis and key immunometabolic enzymes. **Methods:** Thirty-two male Wistar rats were divided into healthy and diabetic groups and treated with either saline or metformin (200 mg/kg) for 10 weeks. Type 2 diabetes was induced by multiple low doses of streptozotocin (30 mg/kg, intraperitoneally) and high-fat diet. Renal function indices, lipid profile, inflammatory cytokines, succinate dehydrogenase (SDH), ATP-citrate lyase (ACLY), and senescence markers were measured, while FABP4 and FOXO1 expression, macrophage infiltration, and kidney histology were assessed using immunoassays and microscopy. **Results:** Metformin considerably reduced serum creatinine, urea, and blood urea nitrogen; normalized the lipid profile; suppressed interleukin (IL)-6 and tumor necrosis factor-α; and increased IL-10 levels. Additionally, it reversed DN-associated alterations in SDH and ACLY; downregulated FABP4, FOXO1, and P16^INK4a^; decreased macrophage infiltration; promoted M2 polarization; and improved renal architecture. **Conclusions:** This study is the first to demonstrate that metformin mitigates diabetic renal senescence by simultaneously targeting the FABP4/FOXO1 axis and immunometabolic enzymes SDH and ACLY. These findings highlight the translational significance of metformin as a prototype for immunometabolic and immunosenescence-directed therapies in DN.

## 1. Introduction

The biological process of senescence causes cells to stop growing, ultimately resulting in their death. Therefore, it is considered an antagonistic hallmark in the aging process [1]. Senescent cells produce proteins known as senescence-associated secretory phenotypes, which include extracellular remodeling factors, chemokines, growth factors, and inflammatory cytokines [2]. In healthy individuals, senescence stops malignant cells from multiplying, causing their growth cycle to halt—a process initiated by cells that are aging or malfunctioning [3].

Diabetes, a metabolic disorder, disrupts normal cell function, including proteins and carbohydrates processing and transport, resulting in premature cellular senescence [4]. One of the complications of diabetes, diabetic nephropathy (DN), manifests when the kidneys are damaged as a result of hyperglycemia. DN is regarded as being the primary cause of kidney damage and aging, one of the main features of which is a continuous worsening in the rate of glomerular filtration and persistent albuminuria [5]. Previous research has compared the mortality rates of individuals with and without CKD based on the 10-year cumulative mortality rate. The findings showed that mortality rate increased in individuals with CKD (approximately 31.1%) compared with those without the disease (11.5%), indicating that CKD is a primary cause of mortality in patients with diabetes [6]. The factors driving the process by which cells age and are damaged include reactive oxygen species (ROS), inflammation, and advanced glycation end products (AGEs), resulting in early senescence of the kidneys [7].

Observations have consistently shown that senescence is a key cellular process responsible for aging of the kidneys in DN and CKD onset [8]. When kidneys age due to DN, ROS promote the accumulation of stressors and inflammation within the organ, inducing senescence through distinct pathways. Kidney aging and damage are exacerbated by this accumulation. During this process, kidneys are more susceptible to injury, and their ability to self-repair is compromised. This situation results from imbalances between the apoptosis and the proliferation of cells and senescence-associated secretory phenotypes (SASPs) [7].

Studies have indicated that among individuals with diabetes, hyperglycemia causes macrophages—from which SASPs are secreted—to be recruited, ultimately resulting in inflammation and cellular senescence of the kidneys [9]. A macrophage is a type of white blood cell that engulfs foreign materials and subsequently triggers an immune response [10]. Macrophages are considered to be key in diabetic kidneys that host a persistent low-grade inflammation, and they can be grouped into two categories according to their inflammatory response: the classical M1 and alternative M2 types [11]. Research performed on renal biopsy tissue samples from humans indicates that the M1/M2 ratios vary according to the extent of DN progression. M1 numbers were elevated when the disease was in its preliminary stages, resulting in damage and inflammation, whereas M2 numbers generally became elevated as the disease progressed, functioning to suppress inflammation and induce human renal biopsy tissue repair [12]. Kidneys are damaged by macrophages owing to injuries from glucotoxicity and lipotoxicity, in addition to inflammation [13,14]. Lipotoxicity in DN arises from cellular stress induced by excessive fatty acids due to diabetes-induced dysregulation of lipid metabolism [12]. In macrophages, fatty acid-binding proteins (FABPs) are expressed and upregulated in various organs, including the adipose fatty acid-binding protein, also known as A-FABP, FABP4, or aP2 [15]. Research involving mice has shown that insulin resistance and diabetes improved when FABP4 was deficient, providing additional evidence to support the role of FABP4 in diabetes [16]. FABP4 constitutes an intracellular lipid-binding protein [17] that is abundantly expressed on endothelial cells, adipocytes, and macrophages. In the metabolic and inflammatory processes taking place in targeted cells, it assists with the regulation of glucose and lipid metabolism, while also playing a role in the early diagnosis of DN [16,18].

FABP4 upregulation occurs via the forkhead box protein O1 (FOXO1), also known as FKHR (forkhead in rhabdomyosarcoma). FOXO1 expression is specifically observed in insulin-responsive tissues, including those found in the liver, pancreas, skeleton, adipose tissue, and skeletal muscle [19]. It is a transcription factor that contributes substantially to the regulation of macrophage phagocytosis, migration, differentiation, and the activation of inflammation [20]. When FOXO1 is deficient, the migration rate and glycolysis suppression are reduced, making them less capable of controlling the growth of tumors in vitro [21]. Posttranslational alterations like ubiquitination, acetylation, and protein phosphorylation tightly control its activity in macrophages. FOXO1 is a key factor in both aging and age-related diseases, promoting the regulation of cellular metabolism, aging, and longevity. Nevertheless, research into FOXO1’s attenuating effect is ongoing [20,22].

The antidiabetic agent metformin is a biguanide drug utilized for treating type 2 diabetes [23]. According to the findings of a randomized, double-blind, placebo-controlled crossover trial, metformin exhibits both metabolic and non-metabolic effects associated with aging in older individuals [24]. Furthermore, a preclinical study showed that metformin is capable of increasing the life expectancy of the invertebrate *Caenorhabditis elegans* as well as mice, supporting the anti-aging effects of metformin [24]. Such effects result from metformin’s ability to protect against chronic inflammation, deficient mitochondrial function, and DNA damage glycation. Additionally, it facilitates DNA repair, which plays an important role in preventing cancer. Signaling molecules in cells that promote longevity, including the mammalian targets of rapamycin and adenosine monophosphate-activated protein kinase (AMPK), are regulated by metformin. A previous report has indicated that life span is extended by metformin through the activation of AMPK expression [25].

The potential benefits of using metformin as an anti-aging agent have been explored in several clinical trials, including TAME (Targeting Aging with Metformin) and MILES (Metformin in Longevity Study). MILES produced initial evidence supporting the anti-aging effects of metformin, while TAME is ongoing and aims to determine whether six years of metformin use can reduce the incidence of age-related diseases. However, metformin has not yet been approved for anti-aging purposes, and further research—particularly in the context of DN—is warranted [25,26].

In summary, diabetes mellitus is a prevalent disease that accelerates cellular senescence and contributes to kidney injury. Previous studies have indicated that macrophage regulation involves the FABP4 and FOXO1 pathways in an atherosclerosis model [27]; however, these mechanisms have not been confirmed in a DN model, representing a critical gap in the literature. Emerging evidence suggests that metformin may modulate cellular senescence through these pathways, but additional research in both diabetic and non-diabetic groups is required to clarify its role in preventing renal aging. Accordingly, in this study, we aimed to investigate the FABP4/FOXO1 pathway in a streptozotocin-induced DN model to elucidate the mechanisms of metformin by which it potentially delays or prevents early renal senescence. Although the study is primarily focused on diabetes-related kidney aging, its findings may also enhance understanding of renal senescence in non-diabetic individuals, offering valuable insights for future research.

## 2. Results

### 2.1. Effects of Metformin Treatment on Metabolic and DN Biomarkers

#### 2.1.1. Effects of Metformin on Blood Glucose Levels

After inducing diabetes using multiple low doses of streptozotocin (STZ) (30 mg/kg, intraperitoneally) once weekly starting at week 3, rats with or without diabetes were treated with or without metformin (200 mg/kg/day) and monitored for changes in blood glucose levels. The results showed a significant elevation of glucose in the diabetic group compared with the normal rats (*p* < 0.001). Conversely, the metformin-treated diabetic group exhibited a significant reduction in blood glucose levels compared with the diabetic rats (*p* < 0.001), and a similar effect was observed in the metformin-treated rats compared with the normal rats (*p* > 0.01) (Table 1; Table S1A,B).

#### 2.1.2. Effects of Metformin on Body Weight and Kidney-Weight-to-Body-Weight Ratio

The kidney-weight-to-body-weight ratio, an indicator of diabetic renal hypertrophy [28], was significantly increased in the diabetic group compared with the normal group (*p* < 0.001; Table 1). This ratio was significantly decreased in the metformin-treated diabetic group compared with the diabetic group (*p* < 0.001). Regarding body weight, metformin significantly reduced body weight in the diabetic group compared with the diabetic group (*p* < 0.05) (Table S1C–E).

#### 2.1.3. Effects of Metformin on the Levels of Lipid Profile Parameters

Based on the measurement of lipid profile in rat serum samples, total cholesterol levels were significantly increased in the diabetic group compared with the normal group (*p* < 0.001; Table 1) and significantly decreased in the metformin-treated diabetic group compared with the diabetic group (*p* < 0.001) (Table S1F). High-density lipoprotein (HDL) levels were significantly lower in the diabetic group than in the normal group (*p* < 0.05), whereas metformin treatment significantly increased HDL levels compared with the diabetic group (*p* < 0.001) (Table S1G). Low-density lipoprotein (LDL) levels were significantly elevated in the diabetic group compared with the normal group (*p* < 0.001) and significantly decreased in the metformin-treated diabetic group compared with the diabetic group (*p* < 0.001) (Table S1H). Triglyceride levels were significantly higher in the diabetic group than in the normal group (*p* < 0.001) and were significantly reduced in the metformin-treated diabetic group compared with the diabetic group (*p* < 0.001) (Table S1I).

### 2.2. Effects of Metformin on Diabetic Kidney Biomarkers

**Figure 1:**
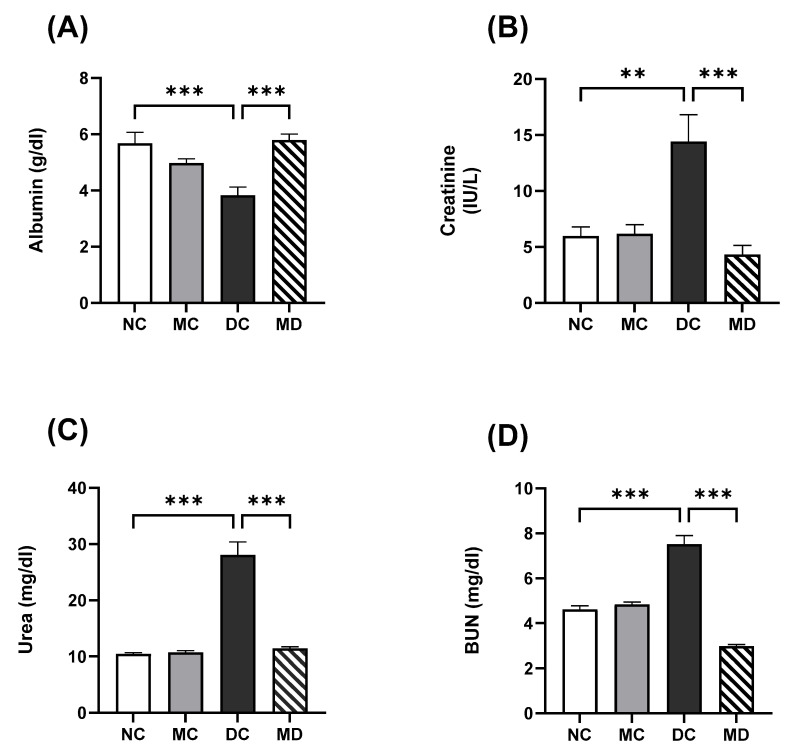
Effects of metformin treatment on nephropathy parameter changes in streptozotocin-induced diabetic rats. Data are expressed as mean ± SEM (n = 8 animals per group). (**A**) Seum albumin level; (**B**) Serum creatinine levels; (**C**) Serum urea levels; and (**D**) Blood urea nitrogen levels. Statistical analyses were performed using a one-way ANOVA followed by a Tukey–Kramer post hoc test; ** *p* < 0.01, *** *p* < 0.001 for MD compared with DC and DC compared with NC, respectively. Abbreviations: NC, normal rats; DC, diabetic rats; MC, metformin-treated rats; MD, metformin-treated diabetic rats; BUN, blood urea nitrogen.

Serum levels of albumin, creatinine, urea, and blood urea nitrogen (BUN) were measured to evaluate changes in renal excretory function (Figure 1). Serum albumin levels were significantly reduced in the diabetic group compared with the normal rats (*p* < 0.001) and were significantly lower in the metformin-treated diabetic group compared with the diabetic group (*p* < 0.001) (Figure 1A). Serum creatinine levels were significantly increased in the diabetic group compared with the normal group (*p* < 0.01) and further decreased in the metformin-treated diabetic group compared with the diabetic group (*p* < 0.001) (Figure 1B). Serum urea and BUN levels were elevated in the diabetic group compared with the normal group (*p* < 0.001), whereas metformin treatment significantly reduced both urea and BUN levels compared with the diabetic group (*p* < 0.01) (Figure 1C,D). No significant changes were observed in kidney biomarkers in metformin-treated rats compared with the normal group, suggesting a protective effect of metformin against DN (Table S1J–M).

### 2.3. Renal Effects of Metformin in Diabetic Rats Evaluated Using Hematoxylin and Eosin- and Periodic Acid–Schiff-Stained Kidney Tissue Sections

**Figure 2:**
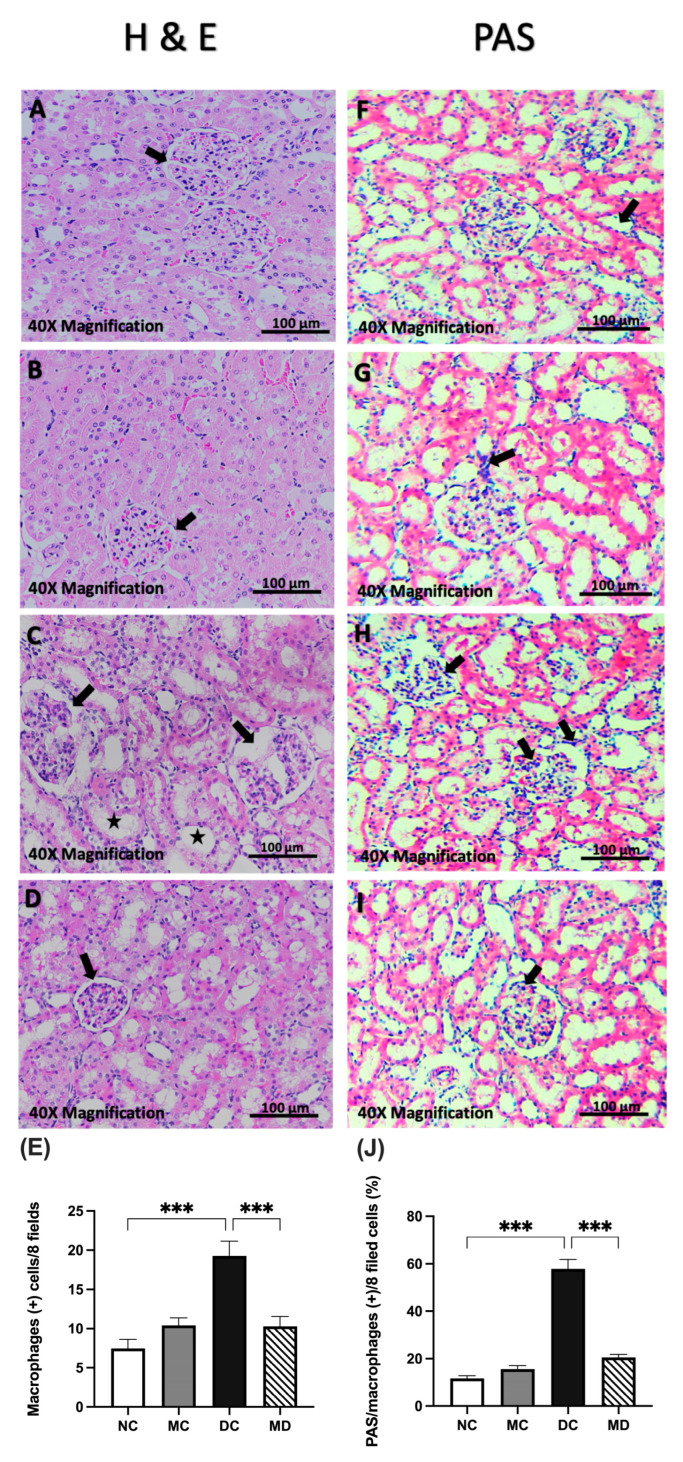
Effect of metformin on the histological morphology of STZ-induced diabetic rat kidneys stained with hematoxylin and eosin (H&E) (**A**–**E**) and periodic acid–Schiff (PAS) (**F**–**J**). ((**A**,**F**), NC) show a normal kidney structure, ((**B**,**G**), MC) display a normal glomerular area (black arrow), ((**C**,**H**), DC) show tubular cell necrosis (asterisk), ((**D**,**I**), MD) show reduced glomerular degradation, and (**E**,**J**) show semi-quantification of macrophage-positive cell analysis. Magnification 40×; scale bar 100 µm. Data are expressed as mean ± SEM (n = 4 rats per group; six fields per kidney section were analyzed per rat). Statistical analyses were performed using a one-way ANOVA followed by a Tukey–Kramer post hoc test; *** *p* < 0.001 for MD compared with DC and DC compared with NC, respectively. Abbreviations: NC, normal rats; DC, diabetic rats; MC, metformin-treated rats; MD, metformin-treated diabetic rats.

Histopathological analysis of kidney tissue showed a normal glomerular area, abundant podocytes, and a healthy mesangial matrix with regular capsular space in the normal group (NC) (Figure 2). In the metformin-treated group (MC), glomerular areas were slightly reduced, with normal tubules compared with the NC. Diabetic untreated rats (DC) exhibited severe degeneration of the mesangial matrix, necrosis, and glomerular expansion. By contrast, metformin-treated diabetic rats (MD) displayed relatively healthy glomeruli, with large podocytes, numerous mesangial cells, and preserved mesangial matrix compared with the DC (Table S1N).

PAS staining revealed normal glomeruli and proximal and distal convoluted tubules without inflammatory changes in the NC. The DC showed accumulation of macrophage-positive cells, enlarged nuclei, and thickening of the basement membrane. Normal rats treated with metformin (MC) were similar to NCs, with normal glomerular structure and fewer macrophage-positive cells. Diabetic rats treated with metformin (MD) exhibited improved mesangial and tubular morphology, increased intact glomeruli, reduced glomerular hypertrophy, decreased necrosis, and fewer macrophage-positive cells (Table S1O).

### 2.4. Metformin Effects on STZ-Induced Changes in the Inflammatory Biomarkers Levels of Experimental Diabetic Rats

**Figure 3:**
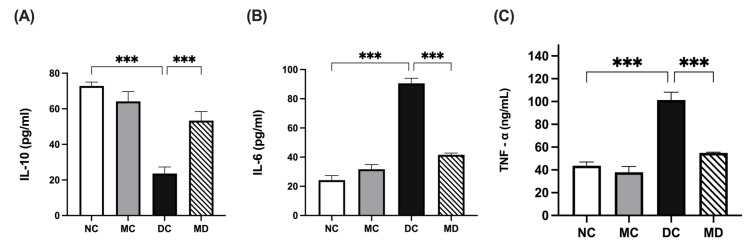
Effects of metformin on plasma inflammatory markers in STZ-induced diabetic rats. Data are expressed as mean ± SEM (n = 8 rats per group). (**A**) Serum IL-10 levels; (**B**) Serum IL-6 levels, and (**C**) Serum TNF-α levels. Statistical analyses were performed using a one-way ANOVA followed by a Tukey–Kramer post hoc test; *** *p* < 0.001 for DC compared with MD and NC compared with DC, respectively. Abbreviations: NC, normal rats; DC, diabetic rats; MC, metformin-treated rats; MD, metformin-treated diabetic rats; IL-10, interleukin-10; IL-6, interleukin-6; TNF-α, tumor necrosis factor alpha.

Plasma interleukin-10 (IL-10) levels were significantly lower in the diabetic group than in the normal group (*p* < 0.001) and significantly increased in the metformin-treated diabetic group compared with the diabetic group (*p* < 0.001) (Figure 3A; Table S1P). Plasma IL-6 levels were significantly higher in the diabetic group than in the normal group (*p* < 0.001) and were significantly decreased in the metformin-treated diabetic group (*p* < 0.001) (Figure 3B; Table S1Q). Plasma tumor necrosis factor-alpha (TNF-α) levels were significantly higher in the diabetic group than in the normal group (*p* < 0.001) and significantly decreased in the metformin-treated diabetic group (*p* < 0.001) (Figure 3C; Table S1R).

### 2.5. Effects of Metformin Treatment on Immunometabolic Markers in Diabetic Rats

**Figure 4:**
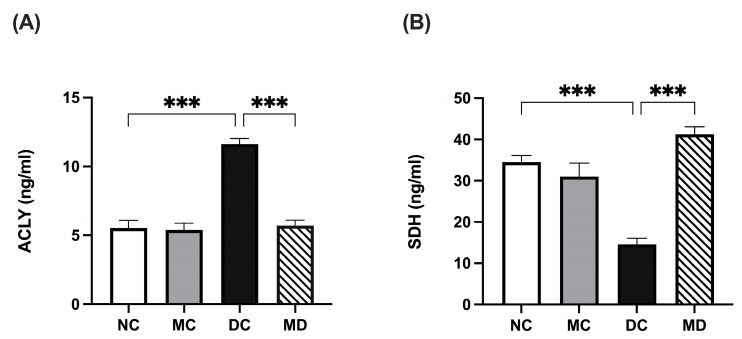
Effects of metformin on kidney immunometabolic markers in STZ-induced diabetic rats. Data are expressed as mean ± SEM (n = 8 rats per group). (**A**) ACLY levels in kidney tissue homogenate; and (**B**) SDH levels in kidney tissue homogenate. Statistical analyses were performed using a one-way ANOVA followed by a Tukey–Kramer post hoc test; *** *p* < 0.001 for DC compared with MD and NC compared with DC, respectively. Abbreviations: NC, normal rats; DC, diabetic rats; MC, metformin-treated rats; MD, metformin-treated diabetic rats; ACLY, ATP citrate lyase; SDH, succinate dehydrogenase.

Kidney ATP citrate lyase (ACLY) levels were significantly higher in the diabetic group than in normal rats (*p* < 0.001), and metformin treatment significantly reduced ACLY levels in diabetic rats (*p* < 0.001), while no significant changes were observed in metformin-treated normal rats (Figure 4A; Table S1S). Succinate dehydrogenase (SDH) activity was unchanged in metformin-treated rats but was significantly reduced in STZ-induced diabetic rats compared with normal rats. Metformin treatment significantly restored SDH activity in diabetic rats (*p* < 0.01) (Figure 4B; Table S1T).

### 2.6. Effects of Metformin Treatment on Kidney Senescence and Macrophages in Rats with Diabetes

**Figure 5:**
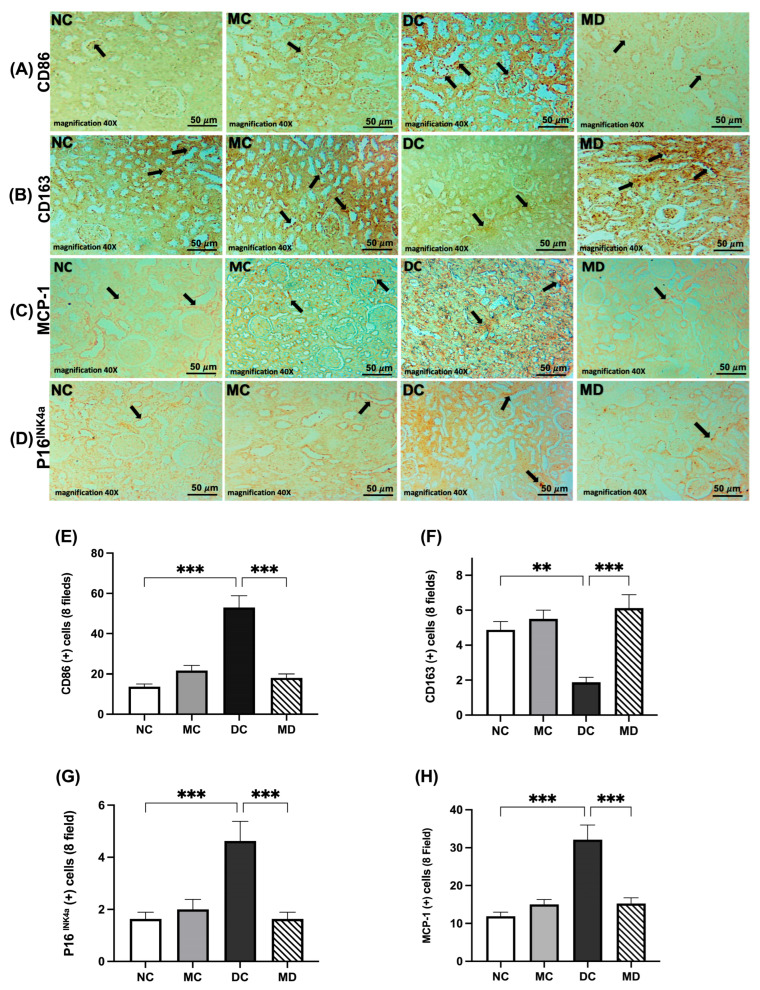
Effects of metformin treatment on CD86, CD163, MCP-1, and p16^INK4a^ expression levels in the kidney tissue of rats with diabetes. Photomicrographs show CD86 (**A**), CD163 (**B**), MCP-1 (**C**), and p16^INK4a^ (**D**) in kidney tissue sections. NC indicates a normal kidney, MC indicates metformin-treated healthy kidney, DC indicates a diabetic kidney, and MD indicates a metformin-treated diabetic kidney. Immunoreactivity is indicated by black arrows. Scale bar 50 µm; magnification 40×. Semi-quantification results for CD86 (**E**), CD163 (**F**), p16^INK4a^ (**G**), and MCP-1 (**H**). Immunoreactivity was analyzed using ImageJ software version 1.8.0_172. Data are expressed as mean ± SEM (n = 4 kidney tissues from 4 rats; six fields per rat). Statistical analyses were performed using a one-way ANOVA followed by a Tukey–Kramer post hoc test; ** *p* < 0.01, *** *p* < 0.001 compared with DC.

To evaluate the potential protective role of metformin on kidney senescence, expressions of p16^INK4a^ and the macrophage phenotype markers cluster of differentiation 86 (CD86), cluster of differentiation 163 (CD163), and monocyte chemoattractant protein-1 (MCP-1) were assessed (Figure 5A,D). CD86 and MCP-1 were significantly higher in the diabetic group than in the normal groups (*p* < 0.01 and *p* < 0.001, respectively) and significantly lower in metformin-treated diabetic rats (*p* < 0.01, *p* < 0.001) (Figure 5A,C,E,H). CD163 levels were lower in diabetic rats (*p* < 0.01) and significantly increased in metformin-treated diabetic rats (*p* < 0.001) (Figure 5B,F). p16^INK4a^ expression was higher in diabetic rats (*p* < 0.001) and significantly reduced by metformin (*p* < 0.001) (Figure 5D,G; Table S1U–X).

### 2.7. Metformin Effects on FABP4 and FOXO1 Expression in Experimental Diabetic Rats

**Figure 6:**
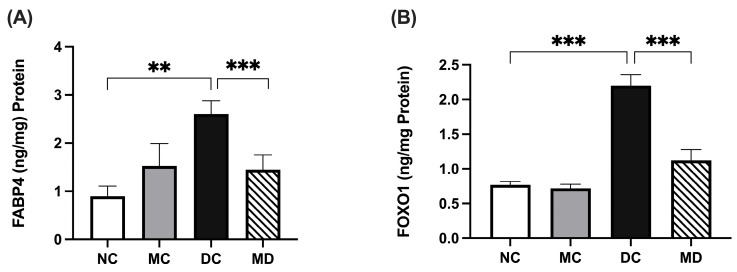
Effects of metformin on FABP4 and FOXO1 kidney tissue levels in STZ-induced diabetic rats. Data are expressed as mean ± SEM (n = 8 rats per group). (**A**) FABP4 levels in kidney tissue homogenate; and (**B**) FOXO1 levels in kidney tissue homogenate. Statistical analyses were performed using a one-way ANOVA followed by a Tukey–Kramer post hoc test; ** *p* < 0.01, *** *p* < 0.001 for DC compared with MD and NC compared with DC, respectively. Abbreviations: NC, normal rats; DC, diabetic rats; MC, metformin-treated rats; MD, metformin-treated diabetic rats.

Kidney FABP4 levels were higher in diabetic rats compared with normal (*p* < 0.001) and were significantly decreased by metformin treatment (*p* < 0.001) (Figure 6A; Table S1Y). Similarly, FOXO1 levels were elevated in diabetic rats (*p* < 0.001) and significantly reduced in metformin-treated diabetic rats (*p* < 0.001) (Figure 6B; Table S1Z).

## 3. Discussion

To the best of our knowledge, this represents the first in vivo experimental study to highlight the role of metformin in attenuating DN through modulation of kidney senescence via the FABP4/FOXO1 pathway. Although FABP4, FOXO1, and metformin have previously been implicated in metabolic regulation and inflammation, no study has attempted to establish a connection between these pathways in relation to diabetes-induced kidney aging. The results presented here contribute novel insights by demonstrating that metformin not only improves renal biomarkers and reduces inflammation but also delays senescence by targeting a key immunometabolic pathway.

To avoid overlap between the disease processes and molecular pathways investigated, we clarify that diabetes represents the primary metabolic disorder inducing systemic hyperglycemia and inflammatory stress; DN refers specifically to kidney injury arising from chronic diabetes; and senescence denotes a distinct cellular program of irreversible growth arrest that is exacerbated–but not defined–by hyperglycemia and DN. In this study, senescence markers (such as P16^INK4a^), inflammatory macrophage phenotypes (CD86, CD163, MCP-1), and immunometabolic enzymes (SDH, ACLY) were evaluated as downstream mechanistic responses to DN rather than as diagnostic criteria for diabetes or DN themselves. This separation ensures that the interpretation of our findings reflects the hierarchical relationship between diabetes, DN development, and the molecular pathways contributing to renal aging.

One of the primary complications of diabetes is DN, ultimately diagnosed in approximately 50% of individuals with type 2 diabetes, making it the leading cause of CKD and end-stage renal failure worldwide [29,30]. This substantial burden underscores the critical need for novel therapeutic strategies that go beyond classical glucose-lowering approaches. By showing that metformin reduces P16^INK4a^ expression, shifts macrophage polarization toward an anti-inflammatory phenotype, and downregulates both FOXO1 and FABP4, this study provides a mechanistic rationale for incorporating metformin into anti-aging strategies in DN. Notably, the macrophage-senescence relationship is a key contributor to DN development. Macrophages induce kidney damage via glucotoxicity; elevated glucose triggers inflammation, which in turn promotes renal senescence [12,13]. Prior studies have demonstrated that targeting macrophages and limiting their recruitment can attenuate DN [12]. Currently, the only approved treatment for DN is renin-angiotensin system blockade, which, despite demonstrated efficacy, has several limitations and provides modest patient benefit [31]. Thus, the discovery of new therapies and molecular targets remains essential.

This study hypothesized that metformin mitigates DN progression by modulating immunosenescence and the FABP4/FOXO1 pathway, which is closely linked to macrophage activation and polarization—key processes in DN progression. Increased FABP4 expression in macrophages enhances proinflammatory signaling and promotes M1 polarization (CD86), whereas FOXO1 regulates macrophage migration, differentiation, and inflammatory activation [15,20]. Suppression of this axis favors an anti-inflammatory M2 phenotype (CD163), associated with tissue repair [12,32]. Uncontrolled activation of FABP4/FOXO1 also contributes to early cellular aging, as indicated by upregulated senescence markers such as P16^INK4a^ in kidney tissue [33,34]. Collectively, these findings provide a robust mechanistic rationale for investigating the FABP4/FOXO1 pathway in relation to macrophage dynamics and senescence in diabetic kidney disease, supporting the relevance of our experimental model. By including SDH and ACLY, this study also identifies how metformin modulates innate immune signaling in DN. Succinate accumulation in proinflammatory macrophages drives hypoxia inducible factor alpha (HIF-1α)-dependent interleukin-1 beta (IL-1β) production, with SDH activity central to this feed-forward loop; restraining succinate/SDH signaling blunts IL-1β output [35]. Concurrently, citrate export fuels ACLY to generate nucleocytosolic acetyl-CoA for histone acetylation and inflammatory gene transcription, a process explicitly driven by TLR ligation in macrophages [21]. Metformin dampens pro-IL-1β production predominantly through AMPK [36], which rewires lipid metabolism and limits acetyl-CoA supply for ACLY-driven chromatin activation [37]. Together, reduced succinate/HIF-1α signaling and constrained ACLY-dependent histone acetylation explain the enzyme-level changes observed in this study and align with the antifibrotic, renoprotective effects detected in diabetic kidneys [38].

Given this proinflammatory environment and its connection to early cellular senescence, strategies targeting macrophage polarization have gained attention. Beyond lowering glucose, metformin exhibits immunomodulatory effects by suppressing M1-driven inflammation and promoting a reparative M2 phenotype, a dual function particularly beneficial in DN, where senescence is accelerated and renal repair is impaired. By simultaneously reducing metabolic dysfunction and constraining inflammation linked to senescence, metformin shows potential as a treatment for aging diabetic kidneys [39,40]. Mechanistically, AMPK activation by metformin suppresses proinflammatory transcriptional programs, including nuclear factor kappa-light-chain-enhancer of activated B cells (NF-kB) and c-Jun N-terminal kinase (JNK)/activating protein-1 (AP-1), which would otherwise promote FABP4 expression and M1 polarization [41,42]. Elevated renal and circulating FABP4 levels are implicated in DN pathogenesis, correlating with fibrosis, proteinuria, and acute kidney injury [43]. Metformin reduces FOXO1 activity, including its translocation and transcriptional output, resulting in coordinated downregulation of FOXO1-dependent immunometabolism genes, including *FABP4* [44,45]. Crosstalk with NAD-dependent protein deacetylase sirtuin-1 (SIRT1) and high-mobility group box 1 (HMGB1) on AMPK further restricts macrophage activation and chemokine production, favoring an anti-inflammatory M2 phenotype and alleviating senescence-associated inflammation [41]. AMPK activation shifts macrophages from glycolysis-dominated, HIF-1α/IL-1β-high M1 states to fatty acid oxidation/oxidative phosphorylation-supported, pro-resolving phenotypes, enhances mitophagy, and suppresses NLRP3 inflammasome activity, dampening IL-1β and overall inflammation [46]. ACLY-dependent histone acetylation is also constrained, slowing M1 polarization [47]. Functionally, metformin reduces pro-IL-1β while increasing IL-10 production in macrophages, consistent with enzyme-level mechanisms [37]. Emerging evidence suggests renoprotective effects of metformin are mediated via the AMPK/SIRT1-FOXO1 pathway, alleviating oxidative stress, enhancing autophagy, and limiting mesangial cell proliferation under hyperglycemia [44]. Collectively, these outcomes provide strong mechanistic support for our hypothesis that metformin attenuates the FABP4/FOXO1 pathway and reduces early renal senescence. To validate these effects, we employed the established STZ plus high-fat diet (HFD) rat model.

STZ, an anti-tumor antibiotic with antineoplastic properties, induces diabetes primarily via ROS-mediated pancreatic cell necrosis, causing hyperglycemia and subsequent kidney damage. STZ can also directly injure the kidneys, complicating assessment of hyperglycemia-induced DN versus STZ toxicity. Therefore, treatment studies should begin only after STZ toxicity subsides, which may take up to three weeks [48,49,50].

In this study, diabetes was induced in male Wistar rats using STZ and a high-fat diet, accurately simulating type 2 diabetes and associated renal complications [51,52]. Findings on kidney biomarkers were consistent with Sanaye et al. [53], showing elevated fasting glucose and kidney-to-body-weight ratio. Damaged kidneys exhibited increased tissue levels of BUN, urea, and creatinine, alongside rising albumin excretion, leading to albuminuria. Hematoxylin and eosin (H&E) and periodic acid–Schiff (PAS) staining revealed necrosis, glomerular expansion, and severe mesangial matrix degeneration, confirming DN [53]. Persistent inflammation elevated M1 (CD86) expression and TNF-α/IL-6 levels, while anti-inflammatory M2 (CD163) and IL-10 levels decreased [54,55,56]. MCP-1, a biomarker of inflammation and tubular damage, was elevated under hyperglycemia, facilitating macrophage recruitment [57,58].

DN is associated with senescence through multiple mechanisms. Diabetes-induced oxidative stress and inflammation shorten telomeres, promoting premature renal senescence via the P53/p21 pathway. DNA damage from ROS, hyperglycemia, and AGEs activates ATM/ATR kinases, triggering P53 and P21 [59]. Senescent kidney cells secrete proinflammatory cytokines, chemokines, growth factors, and matrix remodeling enzymes, sustaining low-grade inflammation, reducing regenerative capacity, and exacerbating renal damage [11].

Renal cellular and immune senescence (“inflammaging”) is accelerated in diabetes, with M1 macrophages driving tubular and endothelial compartment senescence. Metformin mitigates this by activating AMPK, reducing NLRP3/IL-1β signaling, restoring mitochondrial function, and improving tubular cell senescence phenotypes [60]. It reprograms macrophage metabolism via SDH/succinate and ACLY/acetyl-CoA checkpoints, attenuating inflammatory damage and countering immunosenescent remodeling [39].

FABP4 is a key factor in insulin resistance, promoting cholesterol accumulation in macrophages and activating IKK-NF-kB and JNK-AP-1 pathways [15]. Its elevated expression in diabetes is thought to result from early oxidative stress and inflammation, leading to active macrophage accumulation and high serum FABP4 levels [16,61,62]. Renal damage manifests as impaired glomerular filtration and increased tubular reabsorption; urinary FABP4 correlates with renal dysfunction and proteinuria [63,64].

Blocking FABP4 (e.g., with bms309403) benefits metabolic and cardiovascular outcomes in experimental models [16,18]. Based on the findings of other studies, the expression of FABP4 occurs in macrophages, and there is a correlation between higher levels of FABP4 and greater inflammation and metabolic abnormalities [65]. FABP4 promotes macrophage recruitment and activation, resulting in chemokines and proinflammatory cytokines being released [33]. This process induces both inflammation and damage to the kidneys [33]. A previous study involving ischemia–reperfusion-induced acute kidney injury (IRI-AKI) in mice found that increased expression of FABP4 initiates cellular senescence via pathways linked to oxidative stress and lipotoxicity [34].

Disruption of FOXO1 activity is linked to DN progression and cellular senescence through oxidative stress, proinflammatory cytokines, and macrophage regulation. Dysregulation of the FOXO1/FABP4 pathway exacerbates kidney damage, inflammation, and aging [20,66,67]. Our study reinforces this, showing STZ-induced rats with elevated FABP4 and FOXO1 levels.

Metformin demonstrates protective effects in DN, including reduced inflammation, oxidative stress, increased insulin sensitivity, and anti-aging potential (supported by MILES and TAME studies) [26]. Metformin lowered BUN, urea, and creatinine; increased body weight and albumin; and reversed histopathological kidney damage [41,68,69].

It suppresses macrophage activation and infiltration [70]; modulates CD86, CD163, and MCP-1; shifts M1/M2 balance; reduces TNF-α/IL-6; and increases IL-10 [7,55,56,71].

Recent studies indicate aging β-cells influence diabetes progression [72,73]. In rodents, senescent cell accumulation causes glucose intolerance [72], whereas removing p16^Ink4a^-expressing cells restores glucose levels and insulin sensitivity [72]. To the best of our knowledge, this study is the first to evaluate metformin’s effects on p16^INK4a^ [74]. Our observations indicate that the expression of p16^INK4a^ was lowered by metformin, resulting in delayed cellular aging and promoted cell survival. Additionally, evidence suggests that metformin can regulate macrophage function in atherosclerosis through multiple mechanisms, including reducing monocyte differentiation; inhibiting inflammation, oxidative stress, polarization, foam cell formation, and macrophage apoptosis; and acting via AMPK, AMPK-independent targets, NF-κB, ABCG5/8, Sirt1, FOXO1/FABP4, and HMGB1 [27,46]. Furthermore, our study outcomes indicate that metformin reduces FABP4 expression, thereby limiting the nuclear translocation of FOXO1 in atherosclerosis [27,75].

This study also represents the first attempt to explore how metformin affects the FABP4/FOXO1 axis in a diabetic rat model. Remarkable outcomes were observed, showing that the expression of both FABP4 and FOXO1 was lowered by metformin, leading to a reduction in the inflammation linked with aging of the kidneys. In a study by Tronjnar et al. [75], FABP4 was reported to be a key factor in the process by which insulin resistance and diabetes develop [75]. A connection has been established between higher levels of FABP4 and gestational diabetes mellitus and type 2 diabetes mellitus (T2DM), suggesting that its potential as a therapeutic target [75]. Moreover, although we investigated the role of FOXO1 in regulating DN, analogous mechanisms, including apoptosis, inflammation, endothelial dysfunction, and oxidative stress, have been implicated in the development and advancement of diabetic cardiomyopathy (DCM) [6], emphasizing its broader relevance in diabetes complications. Our findings support metformin’s potential to attenuate kidney injury via the FABP4/FOXO1 pathway.

In summary, our results provide new mechanistic insight into metformin’s modulation of immunometabolic pathways in DN. SDH- and ACLY-driven checkpoints act as upstream regulators of macrophage inflammatory responses. Metformin not only activates classical AMPK signaling but also limits succinate accumulation and acetyl-CoA-dependent epigenetic activation, explaining the shift toward anti-inflammatory macrophages and establishing a link between metabolic reprogramming and immunosenescence attenuation in diabetic kidneys.

### Study Limitations and Future Directions

Some limitations of this study are important to acknowledge. First, technical inconsistencies were observed in the Western blot analysis of FABP4 and FOXO1, likely resulting from tissue heterogeneity and protein instability in whole-kidney lysates. However, these issues had minimal impact on our findings, as the pathways were consistently validated using complementary techniques such as biochemical assays, ELISA, and immunohistochemistry. Second, the study was limited to male rats, preventing evaluation of disease progression and response to metformin according to sex. Third, although the FABP4/FOXO1 pathway and immunometabolic enzymes (SDH, ACLY) were identified as primary regulators, pathway-specific inhibition experiments, including pharmacological blocking and genetic knockdown, were not performed, limiting causal verification. Fourth, despite analyses of macrophage infiltration and polarization, understanding of immune heterogeneity could have been improved through high-resolution techniques such as single-cell imaging or flow cytometry. Fifth, we acknowledge that direct assessment of kidney tissue inflammatory cytokines would provide deeper insight into kidney-specific inflammation; therefore, future studies will incorporate kidney tissue inflammatory cytokine profiling and immunohistochemical analyses to validate and extend the current serum-based findings. Finally, because this study was conducted in a rat model, translating these findings to human DN requires further validation. Addressing these limitations through sex-based comparisons and mechanistic studies would enhance the translational potential of our findings for practical applications.

## 4. Materials and Methods

### 4.1. Drugs, Chemicals, and Antibodies

STZ and metformin were obtained from Sigma-Aldrich (St. Louis, MO, USA). Inflammatory biomarkers, namely IL-6 (cat. SEKR-0017), IL-10 (cat. SEKR-0006), and TNF-α (cat. SEKR-0009), were measured using rat-specific ELISA kits manufactured by Solarbio life science (Beijing, China). Rat-specific ELISA kits from Solarbio were also used to assess immunometabolic biomarkers, including SDH (cat. ED-35488), ACLY (cat. ED-36883), and FABP4 (cat. SEKR-0173). Primary antibodies for macrophage phenotypes, such as monoclonal anti-CD163 (cat. ab182422) and monoclonal anti-CD86 (cat. ab220188), were obtained from Abcam^®^ (Biotechnology Inc., Cambridge, UK). Other chemicals and reagents suitable for analytical testing were acquired from standard commercial suppliers.

### 4.2. Experimental Animals

The animals used in the experiments comprised 32 male Wistar albino rats aged 8–10 weeks with weights ranging from 280 to 330 g. All rats were obtained from the Experimental Animal Center of King Saud University, College of Pharmacy, Riyadh, KSA. Appropriate procedures were followed when handling the rats, which were housed in a controlled environment at 21–25 °C with 60% humidity under a 12 h light/dark cycle (12:12 h). Rats were provided with food pellets and tap water and were monitored carefully for one week to allow acclimatization to the laboratory environment prior to the start of the experiment. Procedures were implemented to minimize pain and distress, including careful observation for signs of discomfort, as well as the administration of anesthesia and analgesia. All study protocols adhered to the Experimental Animals Ethics Committee Acts of King Saud University and the guidelines of the Institutional Research Ethics Committee (KSU-SE-24-31). The study regimen was designed based on initial research conducted in various laboratories.

### 4.3. Induction of T2DM

Starting at week 0, 16 rats were fed an HFD to induce T2DM. This was followed by three intraperitoneal (i.p.) injections of 30 mg/kg STZ, administered weekly beginning at week 3 [76,77,78,79]. The STZ solution was prepared by dissolving STZ in 0.1 M cold sodium citrate buffer, adjusted to pH 4.5. Glucose levels were measured 72 h after STZ administration using tail vein blood samples and a Contour TS blood glucose monitoring system (Roche Diagnostic, Indianapolis, IN, USA). Rats were considered diabetic if their random blood glucose level exceeded 13.9 mmol/L (250 mg/dL) [77] and were then selected for further experimentation. The remaining 16 rats were allocated to the normal group and received a standard chow diet along with physiological saline or metformin orally for the entire 10-week treatment period.

### 4.4. Experimental Design

**Figure 7:**
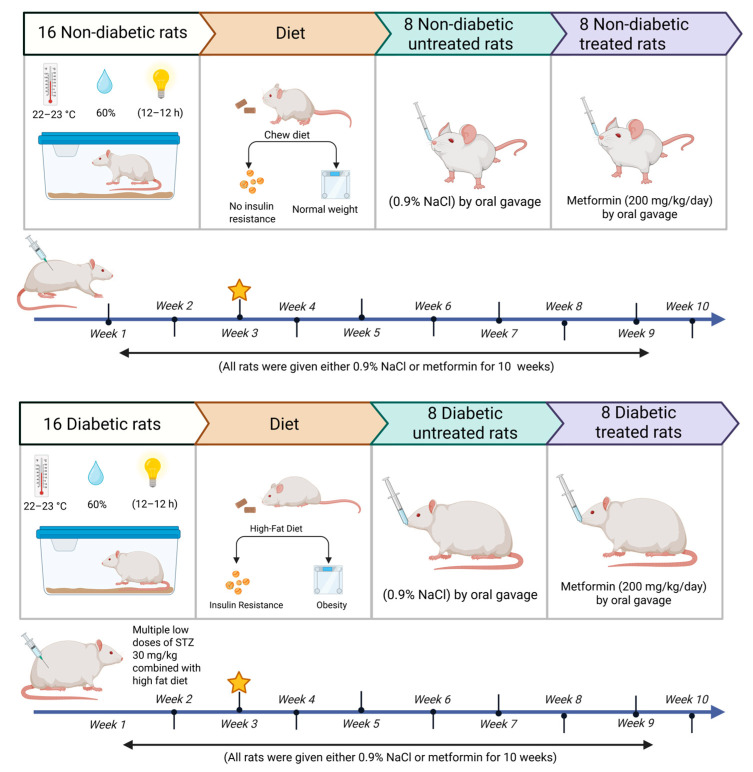
Graphical abstract of the experimental design. Created in BioRender. A, L (2025) https://BioRender.com/9utw7vy (accessed on 27 November 2025). The yellow star denotes the time of diabetes induction.

Sixteen each of normal and diabetic rats were assigned to four groups (n = 8) as described here (Figure 7):

Group 1 (normal control, NC): rats without diabetes administered physiological saline via oral gavage from weeks 0 to 10.

Group 2 (metformin control, MC): rats without diabetes administered metformin (200 mg/kg/day) [80] in distilled water via oral gavage from weeks 0 to 10.

Group 3 (diabetic control, DC): rats with diabetes administered physiological saline via oral gavage from weeks 0 to 10.

Group 4 (diabetic + metformin, MD): rats with diabetes administered metformin (200 mg/kg/day) in distilled water via oral gavage from weeks 0 to 10.

Group sizes were selected based on the researchers’ experience and previous studies [81,82], as this size is considered appropriate from both ethical and scientific perspectives. Using such group sizes in a type 2 diabetes model, especially when animals exhibit variable responses to STZ [83,84], provides adequate statistical power and ensures that study outcomes are reliable and sensitive.

Serum glucose and body weight were measured weekly throughout the study. At the conclusion of the experiment, rats were euthanized using gradual CO_2_ exposure, with a maximum displacement rate of 30% (~51/min), and death was confirmed by the absence of reflexes. Trunk blood was collected, and serum was separated for biochemical analysis. Kidneys were immediately excised, weighed, and immersed in ice-cold phosphate-buffered saline (PBS, 10% *w*/*v*). The kidney-weight-to-body-weight ratio (KW:BW) was calculated to assess DN. Kidney samples were homogenized in ice-cold PBS (10% *w*/*v*) and analyzed using rat-specific ELISA kits for oxidative stress, inflammation, and kidney biomarkers. Selected kidney tissues were fixed in 4% neutral buffered formalin for histological and immunochemical processing, while remaining tissues were stored at −80 °C for molecular analysis.

### 4.5. Biochemical and Molecular Analysis

#### 4.5.1. Determination of Serum Glucose Levels

Glucose was measured using an analytical assay kit (Ray Biotech, Peachtree Corners, GA, USA; Cat no. MA-GLU-1) according to the manufacturer’s instructions.

#### 4.5.2. Determination of DN Biomarkers

Kidney biomarkers (creatinine, urea, BUN, and albumin) were measured in serum using commercial rat kits (created by the United Design Industry (UDI), Hannover, Germany) following manufacturer protocols. Assays involved adding 1 mL of working reagent to 10 µL of each standard, incubating for 5 min at 37 °C, and measuring absorbance at 630 and 340 nm with a spectrophotometer (Biochrom Ltd., Cambridge, UK). Albumin, creatinine, urea, and BUN concentrations were calculated using standard formulas in mg/dL.

#### 4.5.3. Measurement of Lipid TG, Total Cholesterol, and HDL Profiles

Total lipids, cholesterol, triglycerides, and HDL in serum were measured using a spectrophotometer (Biochrom alts, Cambridge, UK) with commercial kits (QUÍMICA Clinica Aplicada, Amposta, Spain). One milliliter of working reagent was added to each quivet (sample, standard, blank), followed by 100 μL of sample solution and thorough mixing. Quivets were incubated at 25 °C for 5 min or 37 °C for 10 min. Readings were taken at 430 nm, The results were calculated to a known factor such that total lipids, cholesterol, and HDL (U/L) concentrations could be determined [85,86].

#### 4.5.4. Determination of Inflammatory Markers

Inflammatory cytokines (IL-6, IL-10, and TNF-α) were quantified using rat-specific ELISA kits (SolarBio^®^, Fengtai, Beijing, China), following the manufacturer’s instructions. Briefly, 96-well plates precoated with specific monoclonal antibodies were used, and absorbance was measured with a microplate reader (BioTek Instruments, Winooski, VT, USA), thus enabling the quantification of cytokine concentration.

#### 4.5.5. Determination of Immunometabolic Markers

ACLY and SDH levels in kidney homogenates were assessed using ELISA kits following manufacturer protocols. Samples (50 μL) and standards were added to wells with 100 μL enzyme conjugate reagent, incubated for 60 min at 37 °C, and washed 4 times. Subsequently, 50 μL of Substrate A + 50 μL of Substrate B were added and incubated at 37 °C for 15 min in the dark. The last step comprised adding 50 μL of the stop solution to every well to prevent the reaction from progressing. Subsequently, absorbance was measured at 450 nm with a microplate reader (BioTek Instruments, Winooski, VT, USA). Sample concentrations were determined based on a standard curve generated following the instructions included in the assay kit.

#### 4.5.6. Quantification of FOXO1 and FABP4 Protein Expression Levels Using ELISA

FOXO1 and FABP4 levels were measured in serum samples using ELISA kits according to the manufacturer’s instructions. For the assays, 50 μL of each standard or sample was incubated in the ELISA plates for 1 h at 37 °C, followed by the addition of 50 μL of the specific primary antibody to each well and a further incubation for 1 h at 37 °C. A secondary antibody was then applied to each well and incubated for 1 h at 37 °C. Subsequently, 100 μL of substrate was added to each well and incubated for 30 min at 37 °C in the dark. Finally, 100 μL of stop solution was added, and absorbance was measured at 450 nm using a microplate reader (BioTek Instruments, Winooski, VT, USA). Sample concentrations were calculated from a standard curve generated according to the assay kit instructions.

#### 4.5.7. Histological Examination

Rats were euthanized by gradually increasing CO_2_ levels, followed by decapitation. Kidneys were immediately excised, washed in ice-cold saline to remove fat and connective tissue, and dissected. Tissues were fixed in 4% neutral buffered formalin for 24 h, dehydrated in graded ethanol, cleared in xylene, and embedded in paraffin. For morphological assessment, 4-µm sections were stained with H&E, Masson’s trichrome, and PAS, with images evaluated by blinded experts.

#### 4.5.8. Immunohistochemistry

Paraffin-embedded kidney sections were used to detect the senescence marker p16^INK4a^, while macrophage infiltration was assessed using CD86 (M1), CD163 (M2), and MCP-1 markers. Immunostaining was performed following previous protocols using the ImmunoCruz ABC system (Santa Cruz, Dallas, TX, USA) [87]. Sections were incubated for 5 min to block non-specific antibody binding, followed by overnight incubation at 4 °C with primary antibodies. Sections were then washed three times in Tris buffer and incubated with biotinylated anti-rabbit IgG (1:100) for 30 min. After washing, the sections were incubated with a diaminobenzidine substrate working solution. To preserve staining, slides were mounted using a mixture of distyrene, plasticizer, and xylene. Tissue sections were examined using a bright-field light microscope (DMRBE; Leica, Bensheim, Germany) equipped with a video camera (ProgRes; Kontron Instruments, Watford, UK), and ImageJ software (version 1.54 g) was used for image viewing and analysis.

### 4.6. Statistical Analysis

Data are reported as means ± standard errors of the mean (SEMs). Comparisons between groups were performed using one-way analysis of variance, followed by the Tukey–Kramer post hoc test. Statistical analysis was conducted using Prism Windows software (Version 9.5.1, GraphPad Software Inc., San Diego, CA, USA), with a *p* value of <0.05 considered statistically significant.

## 5. Conclusions

Our study demonstrated the protective effects of metformin against diabetic nephropathy by the following two key mechanisms: reprogramming macrophage metabolism through SDH- and ACLY-driven pathways and attenuating inflammation-induced senescence. By linking enzyme-level metabolic regulation with renal aging, these findings indicate that metformin provides therapeutic benefits beyond glycemic control. Specifically, the results suggest that metformin may serve as a prototype for immunometabolic interventions that combine metabolic reprogramming with senescence modulation in the diabetes-related aging of organs. Furthermore, this study provides a foundation for future translational research aimed at developing macrophage-targeted immunometabolic and immunosenescence-directed therapies for diabetic kidney disease.

## Figures and Tables

**Table 1:** Effects of metformin treatment on metabolic parameter changes in streptozotocin-induced diabetic rats.

Parameters/Groups	NC	MC	DC	MD
**Initial Glucose (mg/dL)**	102.1 ± 1.20	106.0 ± 2.30	104.4 ± 1.29	104.1 ± 1.59
**Final Glucose (mg/dL)**	70.35 ± 7.28	39.19 ± 4.09 ^##^	343.0 ± 27.94 ^$$$^	62.72 ± 5.67 ***
**KD/BW (mg/g)**	3.15 ± 0.17	4.12 ± 0.27	6.99 ± 0.14 ^$$$^	3.60 ± 0.23 ***
**Initial BW (g)**	286.1 ± 9.68	260.4 ± 5.93	291.4 ± 7.48	258.8 ± 6.73 *
**Final BW (g)**	172.6 ± 11.39	181.1 ± 3.02	235.6 ± 4.20 ^$$$^	208.7 ± 7.96 ***
**Total Cholesterol (mg/dL)**	53.13 ± 4.38	44.00 ± 5.36	116.4 ± 6.95 ^$$$^	49.14 ± 3.74 ***
**HDL (mg/dL)**	33.74 ± 2.93	33.04 ± 2.44	19.72 ± 1.25 ^$^	39.89 ± 4.34 ***
**LDL (mg/dL)**	34.00 ± 2.58	32.00 ± 4.29	122.0 ± 23.65 ^$$$^	39.13 ± 1.63 ***
**TG (mg/dL)**	64.84 ± 4.23	53.60 ± 2.74	186.2 ± 13.94 ^$$$^	69.64 ± 3.09 ***

Values are presented as mean ± SEM (n = 8). A group comparison was performed using a one-way analysis of variance (ANOVA) followed by a Tukey–Kramer post hoc test. For primary comparisons: MD compared with DC: * *p* < 0.05, *** *p* < 0.001; DC compared with NC: ^$^ *p* < 0.05, ^$$$^ *p* < 0.001. For additional comparisons: MC compared with NC ^##^ *p* < 0.01. Abbreviations: NC, normal rats; MC, metformin-treated rats; DC, diabetic rats; MD, metformin-treated diabetic rats; KW/BW ratio, kidney-weight-to-body-weight ratio; HDL, high-density lipoprotein; LDL, low-density lipoprotein; TG: triglyceride.

## Data Availability

The raw data supporting the conclusions of this article will be made available by the authors on request.

## Supplementary Materials

The following supporting information can be downloaded at: https://www.mdpi.com/article/10.3390/ph18121834/s1, Table S1: Original data for all biochemical and molecular parameters.

## Abbreviations

The following abbreviations are used in this manuscript:

DN	Diabetic nephropathy
FABP4	Fatty acid-binding protein 4
FOXO1	Forkhead box protein O1
SDH	Succinate dehydrogenase
ACLY	ATP-citrate lyase
IL	Interleukin
BUN	Blood urea nitrogen
CKD	Chronic kidney disease
ROS	Reactive oxygen species
AGEs	Advanced glycation end products
SASPs	Senescence-associated secretory phenotypes
A-FABP	Adipose fatty acid-binding protein
T2DM	Type 2 diabetes mellitus
STZ	Streptozotocin
HFD	High-fat diet
NC	Normal control
MC	Metformin control
DC	Diabetic control
MD	Diabetic + metformin
H&E	Hematoxylin and eosin
PAS	Periodic acid–Schiff
TNF-α	Tumor necrosis factor-alpha
CD	Cluster of differentiation
MCP-1	Monocyte chemoattractant protein-1
AMPK	Adenosine monophosphate-activated protein kinase
TAME	Targeting Aging with Metformin
MILES	Metformin in Longevity Study
NF-kB	Nuclear factor kappa-light-chain-enhancer of activated B cells
AP-1	Activating protein-1
SIRT1	NAD-dependent protein deacetylase sirtuin-1
HMGB1	High-mobility group box 1
KW:BW	Kidney-weight-to-body-weight ratio
PBS	Phosphate-Buffered Saline
i.p.	Intraperitoneally
ELISA	Enzyme-linked immunosorbent assay
TG	Triglycerides
HIF-1α	Hypoxia inducible factor alpha
IL-1β	Interleukin-1 beta
ABC	ATP-binding cassette
P53	Tumor protein p53

## References

[1] López-Otín C., Blasco M.A., Partridge L., Serrano M., Kroemer G. (2023). Hallmarks of aging: An expanding universe. Cell.

[2] Zhou B., Wan Y., Chen R., Zhang C., Li X., Meng F., Glaser S., Wu N., Zhou T., Li S. (2020). The emerging role of cellular senescence in renal diseases. J. Cell. Mol. Med..

[3] McHugh D., Gil J. (2018). Senescence and aging: Causes, consequences, and therapeutic avenues. J. Cell Biol..

[4] Burton D.G.A., Faragher R.G.A. (2018). Obesity and type-2 diabetes as inducers of premature cellular senescence and ageing. Biogerontology.

[5] Rout P., Jialal I. (2025). Diabetic nephropathy. StatPearls.

[6] Shafaati T., Gopal K. (2024). Forkhead box O1 transcription factor; a therapeutic target for diabetic cardiomyopathy. J. Pharm. Pharm. Sci..

[7] Guo J., Zheng H.J., Zhang W., Lou W., Xia C., Han X.T., Huang W.J., Zhang F., Wang Y., Liu W.J. (2020). Accelerated kidney aging in diabetes mellitus. Oxid. Med. Cell. Longev..

[8] Schmitt R., Susnik N., Melk A. (2015). Molecular aspects of renal senescence. Curr. Opin. Organ. Transplant..

[9] Prattichizzo F., De Nigris V., Mancuso E., Spiga R., Giuliani A., Matacchione G., Lazzarini R., Marcheselli F., Recchioni R., Testa R. (2018). Short-term sustained hyperglycaemia fosters an archetypal senescence-associated secretory phenotype in endothelial cells and macrophages. Redox Biol..

[10] Hirayama D., Iida T., Nakase H. (2017). The phagocytic function of macrophage-enforcing innate immunity and tissue homeostasis. Int. J. Mol. Sci..

[11] Chi M., Tian Z., Ma K., Li Y., Wang L., Nasser M.I., Liu C. (2022). The diseased kidney: Aging and senescent immunology. Immun. Ageing.

[12] Li H.D., You Y.K., Shao B.Y., Wu W.F., Wang Y.F., Guo J.B., Meng X.M., Chen H. (2022). Roles and crosstalks of macrophages in diabetic nephropathy. Front. Immunol..

[13] Bertelli P.M., Pedrini E., Hughes D., McDonnell S., Pathak V., Peixoto E., Guduric-Fuchs J., Stitt A.W., Medina R.J. (2022). Long term high glucose exposure induces premature senescence in retinal endothelial cells. Front. Physiol..

[14] Baker D.J., Childs B.G., Durik M., Wijers M.E., Sieben C.J., Zhong J., Saltness R.A., Jeganathan K.B., Verzosa G.C., Pezeshki A. (2016). Naturally occurring p16(Ink4a)-positive cells shorten healthy lifespan. Nature.

[15] Jin R., Hao J., Yi Y., Sauter E., Li B. (2021). Regulation of macrophage functions by FABP-mediated inflammatory and metabolic pathways. Biochim. Biophys. Acta Mol. Cell Biol. Lipids.

[16] Furuhashi M., Saitoh S., Shimamoto K., Miura T. (2014). Fatty acid-binding protein 4 (FABP4): Pathophysiological insights and potent clinical biomarker of metabolic and cardiovascular diseases. Clin. Med. Insights Cardiol..

[17] Smathers R.L., Petersen D.R. (2011). The human fatty acid-binding protein family: Evolutionary divergences and functions. Human Genom..

[18] Shaker A.M., Mohamed M.E., Ramzy T., Ali M.I. (2023). Serum fatty acid-binding protein 4 as a biomarker for early detection of diabetic nephropathy in type 2 diabetes. Egypt. J. Intern. Med..

[19] Lu H., Huang H. (2011). FOXO1: A potential target for human diseases. Curr. Drug Targets.

[20] Rong S.J., Yang C.L., Wang F.X., Sun F., Luo J.H., Yue T.T., Yang P., Yu Q., Zhang S., Wang C.Y. (2022). The essential role of FoxO1 in the regulation of macrophage function. BioMed Res. Int..

[21] Yan K., Da T.T., Bian Z.H., He Y., Liu M.C., Liu Q.Z., Long J., Li L., Gao C.Y., Yang S.H. (2020). Multi-omics analysis identifies FoxO1 as a regulator of macrophage function through metabolic reprogramming. Cell Death Dis..

[22] Du S., Zheng H. (2021). Role of FoxO transcription factors in aging and age-related metabolic and neurodegenerative diseases. Cell Biosci..

[23] Corcoran C., Jacobs T.F. (2025). Metformin. StatPearls.

[24] Chen S., Gan D., Lin S., Zhong Y., Chen M., Zou X., Shao Z., Xiao G. (2022). Metformin in aging and aging-related diseases: Clinical applications and relevant mechanisms. Theranostics.

[25] Rybina O.Y., Symonenko A.V., Pasyukova E.G. (2023). Compound combinations targeting longevity: Challenges and perspectives. Ageing Res. Rev..

[26] Mohammed I., Hollenberg M.D., Ding H., Triggle C.R. (2021). A critical review of the evidence that metformin is a putative anti-aging drug that enhances healthspan and extends lifespan. Front. Endocrinol..

[27] Feng X., Chen W., Ni X., Little P.J., Xu S., Tang L., Weng J. (2021). Metformin, macrophage dysfunction and atherosclerosis. Front. Immunol..

[28] Al-Qabbaa S.M., Qaboli S.I., Alshammari T.K., Alamin M.A., Alrajeh H.M., Almuthnabi L.A., Alotaibi R.R., Alonazi A.S., Bin Dayel A.F., Alrasheed N.M. (2023). Sitagliptin mitigates diabetic nephropathy in a rat model of streptozotocin-induced type 2 diabetes: Possible role of PTP1B/JAK-STAT pathway. Int. J. Mol. Sci..

[29] Hoogeveen E.K. (2022). The epidemiology of diabetic kidney disease. Kidney Dial..

[30] Al-Shahrani S.M., Shaher B.M., Alragea Y.M., Ali Alqahtani F.M., Binghamiah A.S.M., Alqahtani M.A.M., Mufrrih S.A. (2025). Prevalence and risk factors of diabetic nephropathy among type 2 diabetes patients in family medicine clinic AFHSR Khamis Mushait. J. Fam. Med. Prim. Care.

[31] Sawaf H., Thomas G., Taliercio J.J., Nakhoul G., Vachharajani T.J., Mehdi A. (2022). Therapeutic advances in diabetic nephropathy. J. Clin. Med..

[32] Wang Y.B., Li T., Wang F.Y., Yao X., Bai Q.X., Su H.W., Liu J., Wang L., Tan R.Z. (2025). The dual role of cellular senescence in macrophages: Unveiling the hidden driver of Age-related inflammation in kidney disease. Int. J. Biol. Sci..

[33] Xiao Y., Shu L., Wu X., Liu Y., Cheong L.Y., Liao B., Xiao X., Hoo R.L., Zhou Z., Xu A. (2021). Fatty acid binding protein 4 promotes autoimmune diabetes by recruitment and activation of pancreatic islet macrophages. JCI Insight.

[34] Wang B., Xu J., Ren Q., Cheng L., Guo F., Liang Y., Yang L., Tan Z., Fu P., Ma L. (2022). Fatty acid-binding protein 4 is a therapeutic target for septic acute kidney injury by regulating inflammatory response and cell apoptosis. Cell Death Dis..

[35] Tannahill G.M., Curtis A.M., Adamik J., Palsson-McDermott E.M., McGettrick A.F., Goel G., Frezza C., Bernard N.J., Kelly B., Foley N.H. (2013). Succinate is an inflammatory signal that induces IL-1β through HIF-1α. Nature.

[36] Postler T.S., Peng V., Bhatt D.M., Ghosh S. (2021). Metformin selectively dampens the acute inflammatory response through an AMPK-dependent mechanism. Sci. Rep..

[37] Kelly B., Tannahill G.M., Murphy M.P., O’Neill L.A.J. (2015). Metformin inhibits the production of reactive oxygen species from NADH:Ubiquinone oxidoreductase to limit induction of interleukin-1β (IL-1β) and boosts interleukin-10 (IL-10) in lipopolysaccharide (LPS)-activated macrophages. J. Biol. Chem..

[38] Wang F., Sun H., Zuo B., Shi K., Zhang X., Zhang C., Sun D. (2021). Metformin attenuates renal tubulointerstitial fibrosis via upgrading autophagy in the early stage of diabetic nephropathy. Sci. Rep..

[39] Zheng Q., Zhao J., Yuan J., Qin Y., Zhu Z., Liu J., Sun S. (2024). Delaying renal aging: Metformin holds promise as a potential treatment. Aging Dis..

[40] Zhang T., Zhou L., Makarczyk M.J., Feng P., Zhang J. (2025). The anti-aging mechanism of metformin: From molecular insights to clinical applications. Molecules.

[41] Sun T., Liu J., Xie C., Yang J., Zhao L., Yang J. (2021). Metformin attenuates diabetic renal injury via the AMPK-autophagy axis. Exp. Ther. Med..

[42] Zhou Y., Ma X.Y., Han J.Y., Yang M., Lv C., Shao Y., Wang Y.L., Kang J.Y., Wang Q.Y. (2021). Metformin regulates inflammation and fibrosis in diabetic kidney disease through TNC/TLR4/NF-κB/miR-155-5p inflammatory loop. World J. Diabetes.

[43] Furuhashi M., Ishimura S., Ota H., Hayashi M., Nishitani T., Tanaka M., Yoshida H., Shimamoto K., Hotamisligil G.S., Miura T. (2011). Serum fatty acid-binding protein 4 is a predictor of cardiovascular events in end-stage renal disease. PLoS ONE.

[44] Ren H., Shao Y., Wu C., Ma X., Lv C., Wang Q. (2020). Metformin alleviates oxidative stress and enhances autophagy in diabetic kidney disease via AMPK/SIRT1-FoxO1 pathway. Mol. Cell. Endocrinol..

[45] Guo X., Li X., Yang W., Liao W., Shen J.Z., Ai W., Pan Q., Sun Y., Zhang K., Zhang R. (2021). Metformin targets FOXO1 to control glucose homeostasis. Biomolecules.

[46] Cui Y., Chen J., Zhang Z., Shi H., Sun W., Yi Q. (2023). The role of AMPK in macrophage metabolism, function and polarisation. J. Transl. Med..

[47] Lauterbach M.A., Hanke J.E., Serefidou M., Mangan M.S.J., Kolbe C.C., Hess T., Rothe M., Kaiser R., Hoss F., Gehlen J. (2019). Toll-like receptor signaling rewires macrophage metabolism and promotes histone acetylation via ATP-citrate lyase. Immunity.

[48] Akbarzadeh A., Norouzian D., Mehrabi M.R., Jamshidi S., Farhangi A., Verdi A.A., Mofidian S.M., Rad B.L. (2007). Induction of diabetes by streptozotocin in rats. Indian. J. Clin. Biochem..

[49] Kraynak A.R., Storer R.D., Jensen R.D., Kloss M.W., Soper K.A., Clair J.H., DeLuca J.G., Nichols W.W., Eydelloth R.S. (1995). Extent and persistence of streptozotocin-induced DNA damage and cell proliferation in rat kidney as determined by in vivo alkaline elution and BrdUrd labeling assays. Toxicol. Appl. Pharmacol..

[50] King G.L., Loeken M.R. (2004). Hyperglycemia-induced oxidative stress in diabetic complications. Histochem. Cell Biol..

[51] Wei M., Ong L., Smith M.T., Ross F.B., Schmid K., Hoey A.J., Burstow D., Brown L. (2003). The streptozotocin-diabetic rat as a model of the chronic complications of human diabetes. Heart Lung Circ..

[52] Mazzocco Y.L., Bergero G., Del Rosso S., Cejas Gallardo Z.M., Canalis A.M., Baigorri R.E., Mezzano L., Mladin J.J., Díaz-Gerevini G.T., Martínez Benavidez C. (2025). A novel mouse model for studying complications related to type 2 diabetes using a medium-fat diet, fructose, and streptozotocin. Sci. Rep..

[53] Sanaye M., Sathyapal G., Kulkarni Y.A. (2022). Effect of Costus pictus per se and in combination with metformin and enalapril in streptozotocin induced diabetic nephropathy in rats. J. Diabetes Metab. Disord..

[54] Zhao Y., Jiang Y., Wang F., Sun L., Ding M., Zhang L., Wu B., Zhang X. (2024). High glucose promotes macrophage switching to the M1 phenotype via the downregulation of STAT-3 mediated autophagy. PLoS ONE.

[55] Nna V.U., Abu Bakar A.B., Zakaria Z., Othman Z.A., Jalil N.A.C., Mohamed M. (2021). Malaysian propolis and metformin synergistically mitigate kidney oxidative stress and inflammation in streptozotocin-induced diabetic rats. Molecules.

[56] Abdulmalek S.A., Balbaa M. (2019). Synergistic effect of nano-selenium and metformin on type 2 diabetic rat model: Diabetic complications alleviation through insulin sensitivity, oxidative mediators and inflammatory markers. PLoS ONE.

[57] Chen J., Liu Q., He J., Li Y. (2022). Immune responses in diabetic nephropathy: Pathogenic mechanisms and therapeutic target. Front. Immunol..

[58] Tesch G.H. (2008). MCP-1/CCL2: A new diagnostic marker and therapeutic target for progressive renal injury in diabetic nephropathy. Am. J. Physiol. Ren. Physiol..

[59] Xiong Y., Zhou L. (2019). The signaling of cellular senescence in diabetic nephropathy. Oxid. Med. Cell. Longev..

[60] Yu S., Cheng Y., Li B., Xue J., Yin Y., Gao J., Gong Z., Wang J., Mu Y. (2020). M1 macrophages accelerate renal glomerular endothelial cell senescence through reactive oxygen species accumulation in streptozotocin-induced diabetic mice. Int. Immunopharmacol..

[61] Wen Y., Crowley S.D. (2020). The varying roles of macrophages in kidney injury and repair. Curr. Opin. Nephrol. Hypertens..

[62] Li G., Yang H., Zhang D., Zhang Y., Liu B., Wang Y., Zhou H., Xu Z.X., Wang Y. (2024). The role of macrophages in fibrosis of chronic kidney disease. Biomed. Pharmacother..

[63] Shi M., Ma L., Fu P. (2020). Role of fatty acid binding Protein 4 (FABP4) in kidney disease. Curr. Med. Chem..

[64] Tanaka M., Furuhashi M., Moniwa N., Maeda T., Takizawa H., Matsumoto M., Sakai A., Higashiura Y., Gocho Y., Koyama M. (2020). Significance of urinary fatty acid-binding protein 4 level as a possible biomarker for the identification of minimal change disease in patents with nephrotic-range proteinuria. BMC Nephrol..

[65] Furuhashi M. (2019). Fatty acid-binding Protein 4 in cardiovascular and metabolic diseases. J. Atheroscler. Thromb..

[66] Wang Y., He W. (2021). Improving the dysregulation of FoxO1 activity is a potential therapy for alleviating diabetic kidney disease. Front. Pharmacol..

[67] Su D., Coudriet G.M., Hyun Kim D., Lu Y., Perdomo G., Qu S., Slusher S., Tse H.M., Piganelli J., Giannoukakis N. (2009). FoxO1 links insulin resistance to proinflammatory cytokine IL-1beta production in macrophages. Diabetes.

[68] Zhang S., Xu H., Yu X., Wu Y., Sui D. (2017). Metformin ameliorates diabetic nephropathy in a rat model of low-dose streptozotocin-induced diabetes. Exp. Ther. Med..

[69] Al Za’abi M., Ali B.H., Al Suleimani Y., Adham S.A., Ali H., Manoj P., Ashique M., Nemmar A. (2021). The effect of metformin in diabetic and non-diabetic rats with experimentally-induced chronic kidney disease. Biomolecules.

[70] Vasamsetti S.B., Karnewar S., Kanugula A.K., Thatipalli A.R., Kumar J.M., Kotamraju S. (2015). Metformin inhibits monocyte-to-macrophage differentiation via AMPK-mediated inhibition of STAT3 activation: Potential role in atherosclerosis. Diabetes.

[71] Song A., Zhang C., Meng X. (2021). Mechanism and application of metformin in kidney diseases: An update. Biomed. Pharmacother..

[72] Lee J.H., Lee J. (2022). Endoplasmic reticulum (ER) stress and its role in pancreatic β-cell dysfunction and senescence in type 2 diabetes. Int. J. Mol. Sci..

[73] Murakami T., Inagaki N., Kondoh H. (2022). Cellular senescence in diabetes mellitus: Distinct senotherapeutic strategies for adipose tissue and pancreatic β cells. Front. Endocrinol..

[74] Safwan-Zaiter H., Wagner N., Wagner K.D. (2022). P16INK4A-more than a senescence marker. Life.

[75] Trojnar M., Patro-Małysza J., Kimber-Trojnar Ż., Leszczyńska-Gorzelak B., Mosiewicz J. (2019). Associations between fatty acid-binding protein 4-A proinflammatory adipokine and insulin resistance, gestational and type 2 diabetes mellitus. Cells.

[76] Ibrahim I.Y., Fatma F.A., Elshymaa A.A.-H., Zahra S.A.-R.S. (2023). Pathophysiological mechanisms of type 2 diabetes mellitus and the effects of metformin treatment in adult male albino rats. Minia J. Med. Res..

[77] Ghasemi A., Jeddi S. (2023). Streptozotocin as a tool for induction of rat models of diabetes: A practical guide. Excli J..

[78] Zhang M., Lv X.Y., Li J., Xu Z.G., Chen L. (2008). The characterization of high-fat diet and multiple low-dose streptozotocin induced type 2 diabetes rat model. Exp. Diabetes Res..

[79] Magalhães D.A.D., Kume W.T., Correia F.S., Queiroz T.S., Allebrandt Neto E.W., Santos M.P.D., Kawashita N.H., França S.A.D. (2019). High-fat diet and streptozotocin in the induction of type 2 diabetes mellitus: A new proposal. An. Acad. Bras. Cienc..

[80] Quaile M.P., Melich D.H., Jordan H.L., Nold J.B., Chism J.P., Polli J.W., Smith G.A., Rhodes M.C. (2010). Toxicity and toxicokinetics of metformin in rats. Toxicol. Appl. Pharmacol..

[81] Al-Rasheed N.M., Al-Rasheed N.M., Bassiouni Y.A., Hasan I.H., Al-Amin M.A., Al-Ajmi H.N., Mahmoud A.M. (2018). Simvastatin ameliorates diabetic nephropathy by attenuating oxidative stress and apoptosis in a rat model of streptozotocin-induced type 1 diabetes. Biomed. Pharmacother..

[82] Lu R., Zheng Z., Yin Y., Jiang Z. (2020). Genistein prevents bone loss in type 2 diabetic rats induced by streptozotocin. Food Nutr. Res..

[83] Premilovac D., Gasperini R.J., Sawyer S., West A., Keske M.A., Taylor B.V., Foa L. (2017). A new method for targeted and sustained induction of type 2 diabetes in rodents. Sci. Rep..

[84] Deeds M.C., Anderson J.M., Armstrong A.S., Gastineau D.A., Hiddinga H.J., Jahangir A., Eberhardt N.L., Kudva Y.C. (2011). Single dose streptozotocin-induced diabetes: Considerations for study design in islet transplantation models. Lab. Anim..

[85] Allain C.C., Poon L.S., Chan C.S., Richmond W., Fu P.C. (1974). Enzymatic determination of total serum cholesterol. Clin. Chem..

[86] Burstein M., Scholnick H.R., Morfin R. (1970). Rapid method for the isolation of lipoproteins from human serum by precipitation with polyanions. J. Lipid Res..

[87] Gu R., Bai J., Ling L., Ding L., Zhang N., Ye J., Ferro A., Xu B. (2012). Increased expression of integrin-linked kinase improves cardiac function and decreases mortality in dilated cardiomyopathy model of rats. PLoS ONE.

