# Supplementary material for: Metformin Mitigates Diabetes-Driven Renal Senescence via Immunomodulation and the FABP4/FOXO1 Axis
Source: Pharmaceuticals (Basel). 2025 Dec 1;18(12):1834. doi: 10.3390/ph18121834 (PMC12735873; doi:10.3390/ph18121834)
Supplement: Supplementary file 1 [file pharmaceuticals-18-01834-s001.zip › pharmaceuticals-3985892-supplementary.pdf]

Table S1 (A). Original data for effects of metformin treatment on Initial Glucose (mg/dl) changes in streptozotocin-induced diabetic rats.

	Normal Control	Metformin Control	Diabetic Control	Metformin Diabetic
Repeat no. 1	97	104	99	108
Repeat no. 2	101	99	109	100
Repeat no. 3	99	112	105	103
Repeat no. 4	100	117	108	100
Repeat no. 5	103	100	100	99
Repeat no. 6	106	110	106	106
Repeat no. 7	105	106	106	105
Repeat no. 8	106	100	102	112

Table S1 (B). Original data for effects of metformin treatment on Final Glucose (mg/dl) changes in streptozotocin-induced diabetic rats.

	Normal Control	Metformin Control	Diabetic Control	Metformin Diabetic
Repeat no. 1	92.81867145	36.26570916	328.8732394	49.91023339
Repeat no. 2	96.76840215	27.6481149	321.8309859	80.96947935
Repeat no. 3	72.3518851	26.75044883	323.2394366	78.81508079
Repeat no. 4	76.84021544	43.62657092	311.971831	79.35368043
Repeat no. 5	45.06283662	58.70736086	303.5211268	52.06463196
Repeat no. 6	62.65709156	52.26211849	319.7183099	38.24057451
Repeat no. 7	77.19928187	30.87971275	297.8873239	56.19389587
Repeat no. 8	39.13824057	37.34290844	536.8852459	66.24775583

Table S1 (C). Original data for effects of metformin treatment on Kidney weight/Body weight (mg/g) ratio changes in streptozotocin-induced diabetic rats.

	Normal Control	Metformin Control	Diabetic Control	Metformin Diabetic
Repeat no. 1	3.75203252	4.706827309	7.287974684	4.019762846
Repeat no. 2	3.512260536	4.648148148	6.588888889	2.96744186
Repeat no. 3	3.632954545	3.304964539	7.689530686	4.130111524
Repeat no. 4	3.171631206	4.97338403	6.808219178	3.416666667
Repeat no. 5	2.213793103	4.761061947	7.341328413	4.079710145
Repeat no. 6	3.038225256	3.316793893	6.80495356	3.69379845
Repeat no. 7	3.071518987	3.103703704	6.588235294	4.166037736
Repeat no. 8	2.862662338	4.206896552	6.880434783	2.362962963

Table S1 (D). Original data for effects of metformin treatment on Initial Body weight (g) changes in streptozotocin-induced diabetic rats..

	Normal Control	Metformin Control	Diabetic Control	Metformin Diabetic
Repeat no. 1	246	249	316	253
Repeat no. 2	261	270	270	215
Repeat no. 3	264	282	277	269
Repeat no. 4	282	263	292	264
Repeat no. 5	319	226	271	276

Repeat no. 6	293	262	323	258
Repeat no. 7	316	270	306	265
Repeat no. 8	308	261	276	270

Table S1 (E). Original data for effects of metformin treatment on Final Body weight (g) changes in streptozotocin-induced diabetic rats..

	Normal Control	Metformin Control	Diabetic Control	Metformin Diabetic
Repeat no. 1	246	249	316	253
Repeat no. 2	261	270	270	215
Repeat no. 3	264	282	277	269
Repeat no. 4	282	263	292	264
Repeat no. 5	319	226	271	276
Repeat no. 6	293	262	323	258
Repeat no. 7	316	270	306	265
Repeat no. 8	308	261	276	270

Table S1 (F). Original data for effects of metformin treatment on Cholesterol (mg/dl) changes in streptozotocin-induced diabetic rats.

	Normal Control	Metformin Control	Diabetic Control	Metformin Diabetic
Repeat no. 1	57.78364116	52.77044855	122.6912929	44.32717678
Repeat no. 2	64.11609499	40.63324538	109.4986807	46.17414248
Repeat no. 3	46.17414248	31.92612137	133.2453826	41.9525066
Repeat no. 4	39.84168865	26.64907652	132.9815303	48.81266491
Repeat no. 5	34.82849604	48.28496042	144.591029	62.53298153
Repeat no. 6	67.54617414	57.51978892	92.87598945	37.73087071
Repeat no. 7	49.34036939	26.38522427	98.1530343	68.07387863
Repeat no. 8	65.4353562	67.81002639	96.83377309	43.53562005

Table S1 (G). Original data for effects of metformin treatment on HDL (mg/dl) changes in streptozotocin-induced diabetic rats.

	Normal Control	Metformin Control	Diabetic Control	Metformin Diabetic
Repeat no. 1	43.04418985	24.22258592	12.60229133	14.23895254
Repeat no. 2	30.76923077	25.85924714	18.49427169	42.22585925
Repeat no. 3	41.898527	36.33387889	20.78559738	34.69721768
Repeat no. 4	40.09819967	35.18821604	23.89525368	33.87888707
Repeat no. 5	17.34860884	28.80523732	22.91325696	53.02782324
Repeat no. 6	30.76923077	37.31587561	19.14893617	46.15384615
Repeat no. 7	32.89689034	31.26022913	21.76759411	49.09983633
Repeat no. 8	33.06055646	45.33551555	18.16693944	45.82651391

Table S1 (H). Original data for effects of metformin treatment on LDL (mg/dl) changes in streptozotocin-induced diabetic rats.

	Normal Control	Metformin Control	Diabetic Control	Metformin Diabetic
Repeat no. 1	29	36	113	39
Repeat no. 2	46	22	117	21
Repeat no. 3	34	23	201	37
Repeat no. 4	33	36	122	42
Repeat no. 5	32	27	239	31
Repeat no. 6	26	24	62	44
Repeat no. 7	28	43	50	32
Repeat no. 8	44	56	72	37

Table S1 (I). Original data for effects of metformin treatment on Triglyceride (mg/dl) changes in streptozotocin-induced diabetic rats.

	Normal Control	Metformin Control	Diabetic Control	Metformin Diabetic
Repeat no. 1	74.96251874	41.97901049	210.4947526	59.97001499
Repeat no. 2	71.96401799	65.96701649	185.0074963	74.96251874
Repeat no. 3	71.96401799	62.96851574	207.1964018	83.95802099
Repeat no. 4	68.96551724	53.97301349	212.2938531	59.97001499
Repeat no. 5	62.96851574	50.97451274	183.2083958	63.56821589
Repeat no. 6	74.96251874	47.97601199	211.3943028	65.96701649
Repeat no. 7	47.97601199	50.97451274	185.9070465	71.06446777
Repeat no. 8	44.97751124	53.97301349	93.85307346	77.66116942

Table S1 (J). Original data for effects of metformin treatment on Urea (mg/dl) parameters changes in streptozotocin-induced diabetic rats.

	Normal Control	Metformin Control	Diabetic Control	Metformin Diabetic
Repeat no. 1	10.21971322	11.08805486	37.60587282	12.08998753
Repeat no. 2	10.15291771	11.48882793	23.97958853	10.88766833
Repeat no. 3	11.2884414	9.952531172	34.66687032	9.685349127
Repeat no. 4	10.6872818	9.885735661	29.12284289	11.75600998
Repeat no. 5	10.08612219	9.752144638	20.90699501	12.29037406
Repeat no. 6	11.2884414	10.62048628	20.43942643	12.15678304
Repeat no. 7	9.351371571	12.4907606	24.58074813	11.15485037
Repeat no. 8	10.62048628	10.35330424	33.39775561	11.55562344

Table S1 (K). Original data for effects of metformin treatment on Albumin (g/dl) parameters changes in streptozotocin-induced diabetic rats..

	Normal Control	Metformin Control	Diabetic Control	Metformin Diabetic
Repeat no. 1	4.463917526	4.25257732	2.768041237	4.809278351
Repeat no. 2	5.211340206	5.546391753	3.546391753	5.510309278
Repeat no. 3	5.505154639	4.907216495	3.597938144	5.469072165
Repeat no. 4	5.128865979	5.556701031	3.551546392	5.737113402
Repeat no. 5	7.881443299	5.118556701	3.484536082	6.396907216
Repeat no. 6	5.06185567	4.664948454	3.577319588	6.25257732
Repeat no. 7	6.809278351	4.932989691	4.798969072	5.551546392

Repeat no. 8	5.345360825	4.829896907	5.324742268	6.649484536

Table S1 (L). Original data for effects of metformin treatment on Creatinine (IU/L) parameters changes in streptozotocin-induced diabetic rats.

	Normal Control	Metformin Control	Diabetic Control	Metformin Diabetic
Repeat no. 1	3.296	3.296	14.832	1.648
Repeat no. 2	6.592	6.592	13.184	4.944
Repeat no. 3	8.24	9.888	18.128	3.296
Repeat no. 4	3.296	8.24	8.24	1.648
Repeat no. 5	3.296	6.592	26.368	4.944
Repeat no. 6	8.24	3.296	8.24	3.296
Repeat no. 7	6.592	4.944	19.776	6.592
Repeat no. 8	8.24	6.592	6.592	8.24

Table S1 (M). Original data for effects of metformin treatment on BUN (mg/dl) parameters changes in streptozotocin-induced diabetic rats.

	Normal Control	Metformin Control	Diabetic Control	Metformin Diabetic
Repeat no. 1	4.457605985	5.174563591	6.354166667	3.142361111
Repeat no. 2	4.738154613	5.36159601	6.423611111	2.829861111
Repeat no. 3	4.332917706	4.644638404	7.03125	2.517361111
Repeat no. 4	4.987531172	4.613466334	9.461805556	3.055555556
Repeat no. 5	3.771820449	4.551122195	8.298611111	3.194444444
Repeat no. 6	5.2680798	4.956359102	7.899305556	3.159722222
Repeat no. 7	4.364089776	4.582294264	6.493055556	2.899305556
Repeat no. 8	4.956359102	4.831670823	8.107638889	3.003472222

Table S1 (N). Original data for effects of metformin treatment on Histopathological analysis: hematoxylin and eosin stain (H&E) parameters changes in streptozotocin-induced diabetic rats.

	Normal Control	Metformin Control	Diabetic Control	Metformin Diabetic
Repeat no. 1	8.982035928	5.769230769	20.98765432	7.272727273
Repeat no. 2	7.185628743	8.974358974	29.62962963	11.51515152
Repeat no. 3	5.389221557	8.333333333	17.28395062	8.484848485
Repeat no. 4	2.994011976	10.25641026	20.37037037	9.090909091
Repeat no. 5	11.37724551	10.8974359	14.81481481	7.878787879
Repeat no. 6	3.592814371	11.53846154	22.22222222	7.272727273
Repeat no. 7	10.17964072	13.46153846	12.96296296	16.96969697
Repeat no. 8	10.17964072	14.1025641	16.04938272	13.93939394

Table S1 (O). Original data for effects of metformin treatment on Histopathological analysis: Periodic acid-Schiff (PAS) parameters changes in streptozotocin-induced diabetic rats.

	Normal Control	Metformin Control	Diabetic Control	Metformin Diabetic
Repeat no. 1	7.772020725	15.46391753	74.86910995	16.7539267
Repeat no. 2	7.772020725	19.07216495	50.78534031	19.89528796
Repeat no. 3	16.06217617	14.43298969	60.20942408	18.32460733
Repeat no. 4	10.88082902	17.01030928	65.44502618	21.46596859
Repeat no. 5	13.98963731	19.07216495	64.92146597	21.46596859

Repeat no. 6	10.88082902	6.701030928	59.68586387	24.08376963
Repeat no. 7	12.43523316	18.55670103	46.59685864	26.70157068
Repeat no. 8	13.98963731	14.43298969	40.31413613	15.70680628

Table S1 (P). Original data for effects of metformin treatment on IL-10 (pg/ml) parameters changes in streptozotocin-induced diabetic rats

	Normal Control	Metformin Control	Diabetic Control	Metformin Diabetic
Repeat no. 1	69.24364932497	86.13234428228	33.77738991462	65.86591033351
Repeat no. 2	67.55477982924	69.24364932497	5.066608487193	27.02191193169
Repeat no. 3	72.62138831644	65.86591033351	35.46625941035	43.91060688901
Repeat no. 4	65.86591033351	42.22173739327	30.3996509231	54.04382386339
Repeat no. 5	75.99912730790	40.53286789754	28.71078142742	59.11043235059
Repeat no. 6	84.44347478655	64.17704083778	16.88869495731	64.17704083778
Repeat no. 7	69.24364932497	70.93251882070	20.26643394877	43.91060688901
Repeat no. 8	77.68799680363	74.31025781217	18.57756445304	69.24364932497

Table S1 (Q). Original data for effects of metformin treatment on IL-6 (pg/ml) parameters changes in streptozotocin-induced diabetic rats

	Normal Control	Metformin Control	Diabetic Control	Metformin Diabetic
Repeat no. 1	33.8402240729724	38.4548000829232	90.7533281956988	46.1457600995079
Repeat no. 2	29.2256480630216	39.9929920862402	81.5241761757972	37.6857040812648
Repeat no. 3	24.6110720530709	27.6874560597047	86.138752185748	43.069376092874
Repeat no. 4	27.6874560597047	43.069376092874	86.138752185748	43.8384720945325
Repeat no. 5	7.69096001658464	29.2256480630216	86.138752185748	39.9929920862402
Repeat no. 6	27.6874560597047	23.0728800497539	84.6005601824311	36.1475120779478
Repeat no. 7	13.8437280298524	16.92011203	96.9060962089665	43.069376092874
Repeat no. 8	29.2256480630216	35.378416076	112.288016242136	43.069376092874

Table S1 (R). Original data for effects of metformin treatment on TNF-α (ng/L) parameters changes in streptozotocin-induced diabetic rats

	Normal Control	Metformin Control	Diabetic Control	Metformin Diabetic
Repeat no. 1	25.8382467460229	17.2254978306819	125.50005	54.137278896429
Repeat no. 2	51.6764934920458	46.7549226832796	78.7451329402603	52.9068861942374
Repeat no. 3	46.7549226832796	45.524529981088	89.8186672599844	56.5980643008121
Repeat no. 4	41.8333518745133	47.9853153854711	119.348092112582	55.3676715986205
Repeat no. 5	34.4509956613639	44.2941372788964	115.656914006007	52.9068861942374
Repeat no. 6	50.4461007898543	29.5294248525976	76.2843475358772	55.3676715986205
Repeat no. 7	51.6764934920458	54.137278896429	92.2794526643675	57.8284570030037
Repeat no. 8	46.7549226832796	18.4558905328735	113.196128601624	54.137278896429

Table S1 (S). Original data for effects of metformin treatment on ACLY (ng/ml) parameters changes in streptozotocin-induced diabetic rats

	Normal Control	Metformin Control	Diabetic Control	Metformin Diabetic
Repeat no. 1	4.3263441823418	7.20031677573244	11.3358833341018	4.86175235284498
Repeat no. 2	3.66169955688957	2.68934908632058	9.7481211732993	3.76631954422927
Repeat no. 3	8.50498955902754	5.12022526274307	13.1267313526814	6.3079698248938
Repeat no. 4	4.59097120914222	5.24946171769211	10.6897010593566	4.74482413170061
Repeat no. 5	6.3079698248938	6.66490860522926	11.3789621524182	6.44336039674518
Repeat no. 6	5.44023934166451	5.63101696563691	12.1236102976008	6.52336391647554
Repeat no. 7	6.64029213761991	5.95718516146068	12.9974948977324	5.92641457694901
Repeat no. 8	4.81867353452863	4.66482061197025	11.6189727116093	7.06492620388106

Table S1 (T). Original data for effects of metformin treatment on SDH (ng/ml) parameters changes in streptozotocin-induced diabetic rats

	Normal Control	Metformin Control	Diabetic Control	Metformin Diabetic
Repeat no. 1	40.0669724174901	24.6374550890156	11.9454327704318	40.3158356002074
Repeat no. 2	34.5919823977088	37.0806142248821	12.1942959531492	40.8135619656421
Repeat no. 3	33.8453928495568	28.3704028297756	21.899960079125	36.3340246767301
Repeat no. 4	40.8135619656421	26.6283605507543	11.6965695877145	40.3158356002074
Repeat no. 5	33.5965296668395	26.8772237334716	11.9454327704318	47.7817310817273
Repeat no. 6	27.8726764643409	51.7635420052046	13.1897486840185	50.2703629089006
Repeat no. 7	29.1169923779276	25.3840446371676	13.5879297763662	38.0760669557514
Repeat no. 8	36.3340246767301	27.3749500989063	20.406780982821	36.0851614940128

Table S1 (U). Original data for effects of metformin treatment on CD86 (+) cells 8 folds immunohistochemistry parameters changes in streptozotocin-induced diabetic rats

	Normal Control	Metformin Control	Diabetic Control	Metformin Diabetic
Repeat no. 1	3 (17%)	6 (37%)	10 (76%)	2 (10%)
Repeat no. 2	3 (15%)	1 (25%)	16 (74%)	4 (19%)
Repeat no. 3	4 (8%)	2 (19%)	4 (33%)	1 (24%)
Repeat no. 4	3 (16%)	8 (16%)	4 (63%)	7 (18%)
Repeat no. 5	1 (8%)	7 (20%)	8 (42%)	1 (15%)
Repeat no. 6	3 (15%)	1 (23%)	4 (40%)	1 (17%)
Repeat no. 7	2 (17%)	3 (13%)	9 (41%)	2 (27%)
Repeat no. 8	3 (14%)	2 (20%)	11 (55%)	4 (15%)

Figure S1 (V). Original data for effects of metformin treatment on CD163 (+) cells 8 folds immunohistochemistry parameters changes in streptozotocin-induced diabetic rats

	Normal Control	Metformin Control	Diabetic Control	Metformin Diabetic
Repeat no. 1	4 (18%)	5 (49%)	1 (21%)	6 (66%)
Repeat no. 2	4 (65%)	5 (48%)	2 (17%)	7 (77%)
Repeat no. 3	6 (70%)	8 (66%)	2 (35v)	3 (72%)
Repeat no. 4	6 (63%)	6 (34%)	3 (33%)	5 (37%)
Repeat no. 5	5 (62%)	5 (41%)	3 (27%)	5 (67%)

Repeat no. 6	7 (59%)	7 (59%)	1 (21%)	5 (87%)
Repeat no. 7	3 (47%)	4 (83%)	2 (15%)	10(73%)
Repeat no. 8	4 (30%)	4 (80%)	1 (25%)	8 (76%)

Table S1 (W). Original data for effects of metformin treatment on P16^{INK4a} (+) cells 8 folds immunohistochemistry parameters changes in streptozotocin-induced diabetic rats

	Normal Control	Metformin Control	Diabetic Control	Metformin Diabetic
Repeat no. 1	1 (10%)	2 (28%)	6 (59%)	1 (13%)
Repeat no. 2	1 (19%)	1 (18%)	2 (60.3%)	2 (12%)
Repeat no. 3	2 (16%)	2 (17%)	5 (42%)	1 (11%)
Repeat no. 4	2 (10%)	1 (12%)	5 (55%)	1 (15%)
Repeat no. 5	3 (17%)	3 (17%)	3 (52%)	3 (15%)
Repeat no. 6	1 (15%)	2 (13%)	2 (52%)	2 (10%)
Repeat no. 7	1 (15%)	4 (23%)	8 (21%)	1 (20%)
Repeat no. 8	2 (12%)	1 (16%)	6 (25%)	2 (15%)

Table S1 (X). Original data for effects of metformin treatment on MCP-1 (+) cells 8 folds immunohistochemistry parameters changes in streptozotocin-induced diabetic rats

	Normal Control	Metformin Control	Diabetic Control	Metformin Diabetic
Repeat no. 1	3 (10%)	3 (11%)	6 (37%)	1 (14%)
Repeat no. 2	1 (8%)	3 (12%)	5 (39%)	1 (20%)
Repeat no. 3	2 (10%)	5 (14%)	4 (40%)	4 (15%)
Repeat no. 4	3 (13%)	2 (22%)	6 (26%)	4 (20%)
Repeat no. 5	4 (13%)	1 (16%)	6 (39%)	1 (16%)
Repeat no. 6	3 (10%)	1 (12%)	6 (15%)	3 (18%)
Repeat no. 7	1 (18%)	5 (17%)	5 (43%)	3 (7%)
Repeat no. 8	1 (13%)	3 (16%)	8 (18%)	5 (12%)

Table S1 (Y) Original data for effects of metformin treatment on FABP4 (ng/mg protein) parameters changes in streptozotocin-induced diabetic rats

	Normal Control	Metformin Control	Diabetic Control	Metformin Diabetic
Repeat no. 1	0.566539333	0.43361302	2.659804276	1.164421868
Repeat no. 2	0.248896615	0.585552392	4.15560168	0.697459372
Repeat no. 3	0.377391116	0.487177882	1.455531792	0.357264569
Repeat no. 4	0.404126479	0.48850716	2.253703449	2.209200663
Repeat no. 5	1.498481358	2.109714577	2.626002628	2.378090996
Repeat no. 6	1.295236243	2.065983895	2.667560079	0.801985577
Repeat no. 7	0.953868998	1.835668948	2.883444962	1.3252013
Repeat no. 8	1.838664226	4.205394562	2.147423326	2.660277865

Table S1 (Z) Original data for effects of metformin treatment on FOXO1 (ng/mg protein) parameters changes in streptozotocin-induced diabetic rats

	**Normal Control**	**Metformin Control**	**Diabetic Control**	**Metformin Diabetic**
Repeat no. 1	0.994727468	0.656683708	1.603201586	0.846600097
Repeat no. 2	0.775980316	0.694751921	2.022085299	0.901745462
Repeat no. 3	0.592803478	0.607546145	2.478159532	0.61486816
Repeat no. 4	0.749745321	0.575084032	1.771741354	1.015088309
Repeat no. 5	0.893721117	0.593437402	1.98467706	1.093547366
Repeat no. 6	0.833363517	0.805051177	2.969230731	1.015428316
Repeat no. 7	0.616188587	0.705208344	2.269265469	2.051577096
Repeat no. 8	0.704872265	1.10659897	2.504997633	1.444421992
